# Aiolos promotes CXCR3 expression on Th1 cells via positive regulation of IFN-**γ**/STAT1 signaling

**DOI:** 10.1172/jci.insight.180287

**Published:** 2024-11-19

**Authors:** Melissa R. Leonard, Devin M. Jones, Kaitlin A. Read, Srijana Pokhrel, Jasmine A. Tuazon, Robert T. Warren, Jacob S. Yount, Kenneth J. Oestreich

**Affiliations:** 1 Department of Microbial Infection and Immunity, The Ohio State University College of Medicine and Wexner Medical Center, Columbus, Ohio, USA.; 2 Combined Anatomic Pathology Residency/PhD Program, The Ohio State University College of Veterinary Medicine, Columbus, Ohio, USA.; 3 Biomedical Sciences Graduate Program and; 4 Medical Scientist Training Program, The Ohio State University College of Medicine, Columbus, Ohio, USA.; 5 Infectious Diseases Institute, The Ohio State University College of Medicine and Wexner Medical Center, Columbus, Ohio, USA.; 6 Pelotonia Institute for Immuno-Oncology, The Ohio State University Comprehensive Cancer Center, Columbus, Ohio, USA.

**Keywords:** Immunology, Infectious disease, Cytokines, T cells, Th1 response

## Abstract

CD4^+^ T helper 1 (Th1) cells coordinate adaptive immune responses to intracellular pathogens, including viruses. Key to this function is the ability of Th1 cells to migrate within secondary lymphoid tissues, as well as to sites of inflammation, which relies on signals received through the chemokine receptor CXCR3. CXCR3 expression is driven by the Th1 lineage-defining transcription factor T-bet and the cytokine-responsive STAT family members STAT1 and STAT4. Here, we identify the Ikaros zinc finger (IkZF) transcription factor Aiolos (*Ikzf3*) as an additional positive regulator of CXCR3 both in vitro and in vivo using a murine model of influenza virus infection. Mechanistically, we found that Aiolos-deficient CD4^+^ T cells exhibited decreased expression of key components of the IFN-γ/STAT1 signaling pathway, including JAK2 and STAT1. Consequently, Aiolos deficiency resulted in decreased levels of STAT1 tyrosine phosphorylation and reduced STAT1 enrichment at the *Cxcr3* promoter. We further found that Aiolos and STAT1 formed a positive feedback loop via reciprocal regulation of each other downstream of IFN-γ signaling. Collectively, our study demonstrates that Aiolos promotes CXCR3 expression on Th1 cells by propagating the IFN-γ/STAT1 cytokine signaling pathway.

## Abstract 2

Immune cells utilize chemokine receptors to migrate to sites of infection. Here, we identify Aiolos, a transcription factor targeted by certain chemotherapeutics, as a positive regulator of the chemokine receptor CXCR3. Our findings are significant as they suggest therapeutically targeting Aiolos could impact immune cell migration.

## Graphical abstract

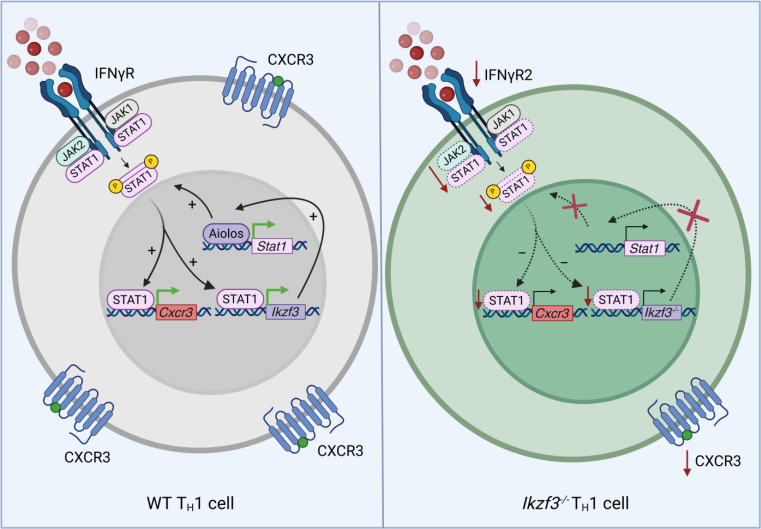

## Introduction

During adaptive immune responses to infection, naive CD4^+^ T cells differentiate into T helper subsets with distinct effector functions. T helper 1 (Th1) cells represent one such subset that produces IFN-γ and directs immune responses against intracellular pathogens. Differentiation of effector subsets is initiated when antigen-presenting cells (APCs) deliver cognate antigen to the T cell receptor (TCR) expressed on the surface of a naive CD4^+^ T cell. Additional signals received in the form of costimulatory molecules and environmental cytokines further propagate T cell activation and differentiation into specific subsets (1–5). As part of this process, signaling through cytokine receptors leads to activation of Janus kinases (JAKs) and ensuing tyrosine phosphorylation of STAT factors, which then dimerize, translocate to the nucleus, and bind to target genes (6–9). For example, IFN-γ signaling via STAT1 and IL-12 signaling via STAT4 both stimulate the expression of T-bet (*Tbx21*), the lineage-defining transcription factor for the Th1 gene program (10–12). In turn, T-bet promotes IFN-γ production via direct transcriptional activation of the *Ifng* gene, thus creating a positive feedback loop that drives Th1 differentiation (13–15).

More recently, Ikaros zinc finger (IkZF) transcription factors have been implicated in the regulation of CD4^+^ T cell programming events (16, 17). IkZF factors contain conserved N-terminal zinc finger (ZF) domains that mediate DNA-binding specificity as well as C-terminal protein interaction domains that enable IkZF dimerization and recruitment of coregulators, such as chromatin remodeling complexes (16–22). The importance of IkZF factors to immune cell function is underscored by studies in humans that have described missense mutations in the IkZF family member Aiolos (*Ikzf3*) that result in immunodeficiency. These changes have been associated with abnormalities in B and T cell differentiation, increased susceptibility to infectious diseases, and elevated risk for certain types of hematological malignancies (23–26). One study specifically identified a heterozygous Aiolos mutation associated with impaired Th cell polarization, which led to reduced numbers of T follicular helper (Tfh) and Th1 cells in affected patients (25).

IkZF factors have also emerged as key regulators of cytokine signaling pathways (18). Previous work from our lab identified a transcription factor complex composed of Aiolos and STAT3 that promoted Tfh cell programming (18). Similarly, we established that a second IkZF/STAT factor complex composed of Eos (*Ikzf4*) and STAT5 drives Th2 differentiation by inducing expression of IL-4 and IL-2 cytokine receptors (27). Further, we demonstrated that Aiolos modulates IL-2 responsiveness via repression of the IL-2Rα (CD25) and IL-2Rβ (CD122) subunits, both promoting Tfh differentiation and suppressing cytotoxic programming of CD4^+^ T cells (28). Beyond our findings, others have found that Aiolos regulates cytokine production, including direct silencing of the *Il2* locus in Th17 cells (29). These collective findings suggest that a complex interplay exists between IkZF factors, cytokine signaling pathways, and Th cell programming events, much of which remains enigmatic.

Like cytokines, chemokines signal through specific receptors and serve as integral mediators of both CD4^+^ T cell differentiation and migration (30–32). The chemokine receptor CXCR3 is a G protein–coupled receptor that is highly expressed on the surface of Th1 cells (33, 34). CXCR3 responds to 3 IFN-inducible ligands (CXCL9, CXCL10, and CXCL11) and directs Th1 cells to sites of inflammation (33, 35, 36). Although Th1 cell responses are beneficial during infection, their activities are typically tightly controlled to prevent destruction of healthy tissue. To this end, aberrant activities of Th1 cells have been implicated in autoimmunity, and therapeutically targeting CXCR3 appears to have disease-specific advantages (35, 37–43). As such, obtaining a better understanding of the transcriptional mechanisms regulating CXCR3 expression may provide additional insight for the development of novel therapeutics.

Here, we identify Aiolos as a positive regulator of CXCR3 expression in both in vitro–generated Th1 cells and those that arise in response to murine influenza virus infection. Mechanistically, we find that Aiolos-deficient CD4^+^ T cells have reduced expression of components of the IFN-γ/STAT1 signaling pathway, which results in decreased STAT1 activation and enrichment at the *Cxcr3* promoter. We further find that *Stat1* is a direct Aiolos target gene, with Aiolos both modulating chromatin accessibility at, and driving activity of, the *Stat1* promoter. Moreover, we demonstrate that Aiolos expression itself is dependent upon IFN-γ signaling and that STAT1 directly binds the *Ikzf3* promoter. Collectively, our findings reveal that Aiolos promotes CXCR3 expression on Th1 cells by propagating IFN-γ/STAT1 signaling via a positive feedback loop with STAT1.

## Results

### CXCR3 expression is reduced on Aiolos-deficient CD4^+^ Th1 cells.

**Figure 1:**
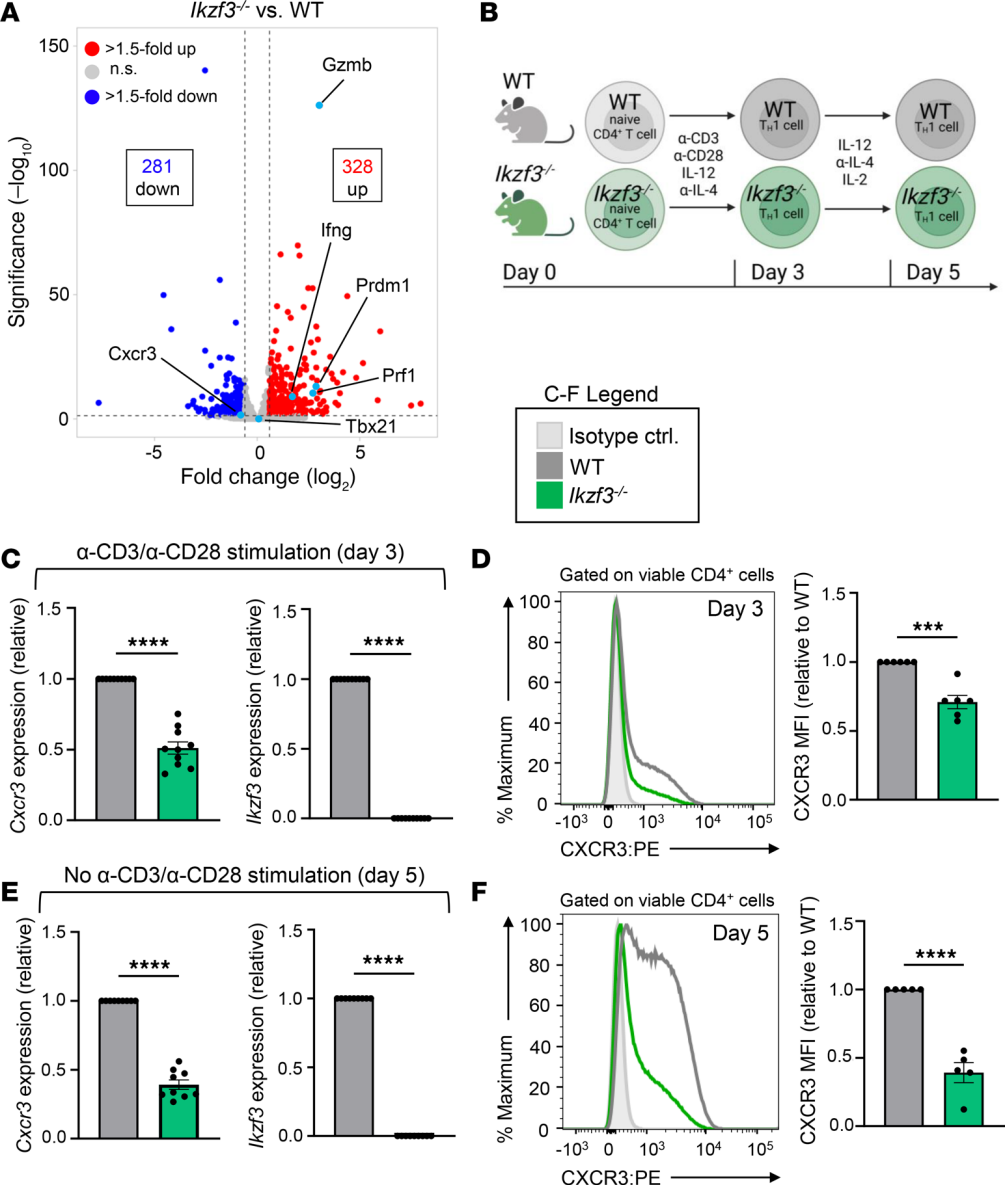
CXCR3 expression is reduced on Aiolos-deficient Th1 cells. (**A**) Published RNA-Seq data (GSE203065) from in vitro–generated WT and *Ikzf3^–/–^* Th1 cells were assessed for differentially expressed genes (DEGs). A volcano plot displays gene expression changes at day 3. Genes are color-coded: no significant changes (gray), upregulated genes with > 1.5-fold change with *P* < 0.05 (red), downregulated genes with > 1.5-fold change with *P* < 0.05 (blue), and selected genes of interest (turquoise). (**B**) Schematic of Th1 cell culturing system. Naive CD4^+^ T cells were stimulated with anti-CD3/CD28 (α-CD3/CD28) under Th1-polarizing conditions (IL-12, α-IL-4). On day 3, cells were harvested or removed from stimulation and placed into fresh Th1 conditions with IL-2 for an additional 2 days. (**C**) At day 3, transcript analysis was performed via quantitative reverse transcription PCR (qRT-PCR). Transcript was normalized to *Rps18* and presented as fold-change compared with WT control (*n* = 10 biological replicates from 10 independent experiments, mean ± SEM; ****P < 0.0001, 2-tailed unpaired Student’s *t* test). (**D**) Representative flow cytometric analysis for CXCR3 on day 3 Th1 cells. Data displayed as median fluorescence intensity (MFI) fold-change compared with WT controls (*n* = 6 biological replicates from 6 independent experiments, mean ± SEM; ***P < 0.001, 2-tailed unpaired Student’s *t* test). (**E**) At day 5, transcript analysis was performed as in **C** (*n* = 9 biological replicates from 9 independent experiments, mean ± SEM; ****P < 0.0001, 2-tailed unpaired Student’s *t* test). Note: *Cxcr3* and *Ikzf3* transcript data presented here are the same as in Figure 5B. (**F**) Representative flow cytometric analysis for CXCR3 on day 5 Th1 cells. Data displayed as MFI fold-change compared with WT controls (*n* = 5 biological replicates from 5 independent experiments, mean ± SEM; ****P < 0.0001, 2-tailed unpaired Student’s *t* test).

We previously reported that Aiolos functions as a repressor of CD4^+^ T cell cytotoxic programming via suppression of IL-2/STAT5 signaling (28). In line with these findings, RNA-Seq analysis of WT versus Aiolos-deficient (*Ikzf3^–/–^*) Th1 cells revealed increased expression of many STAT5 target genes associated with cytotoxic function, including *Gzmb*, *Prf1*, *Ifng*, and *Prdm1* (Figure 1A). In contrast, expression of *Cxcr3* (encoding CXCR3), a chemokine receptor that guides migration of both Th1 cells and CD4^+^ cytotoxic T lymphocytes, was reduced in the absence of Aiolos (Figure 1A). Consistent with the RNA-Seq data, transcript and flow cytometric analyses of in vitro–generated *Ikzf3^–/–^* Th1 cells revealed significant decreases in both CXCR3 transcript and cell surface expression compared with WT after 3 days of differentiation (Figure 1, B–D). In accordance with previous reports describing the inhibition of CXCR3 expression with persistent TCR stimulation, the reduction in cell surface expression of CXCR3 on *Ikzf3^–/–^* Th1 cells was further enhanced after cells were removed from stimulation with α-CD3 and α-CD28 antibodies and cultured for an additional 48 hours (Figure 1, B, E, and F) (44). These findings suggested that CXCR3 expression may be regulated by an Aiolos-dependent mechanism.

**Figure 2:**
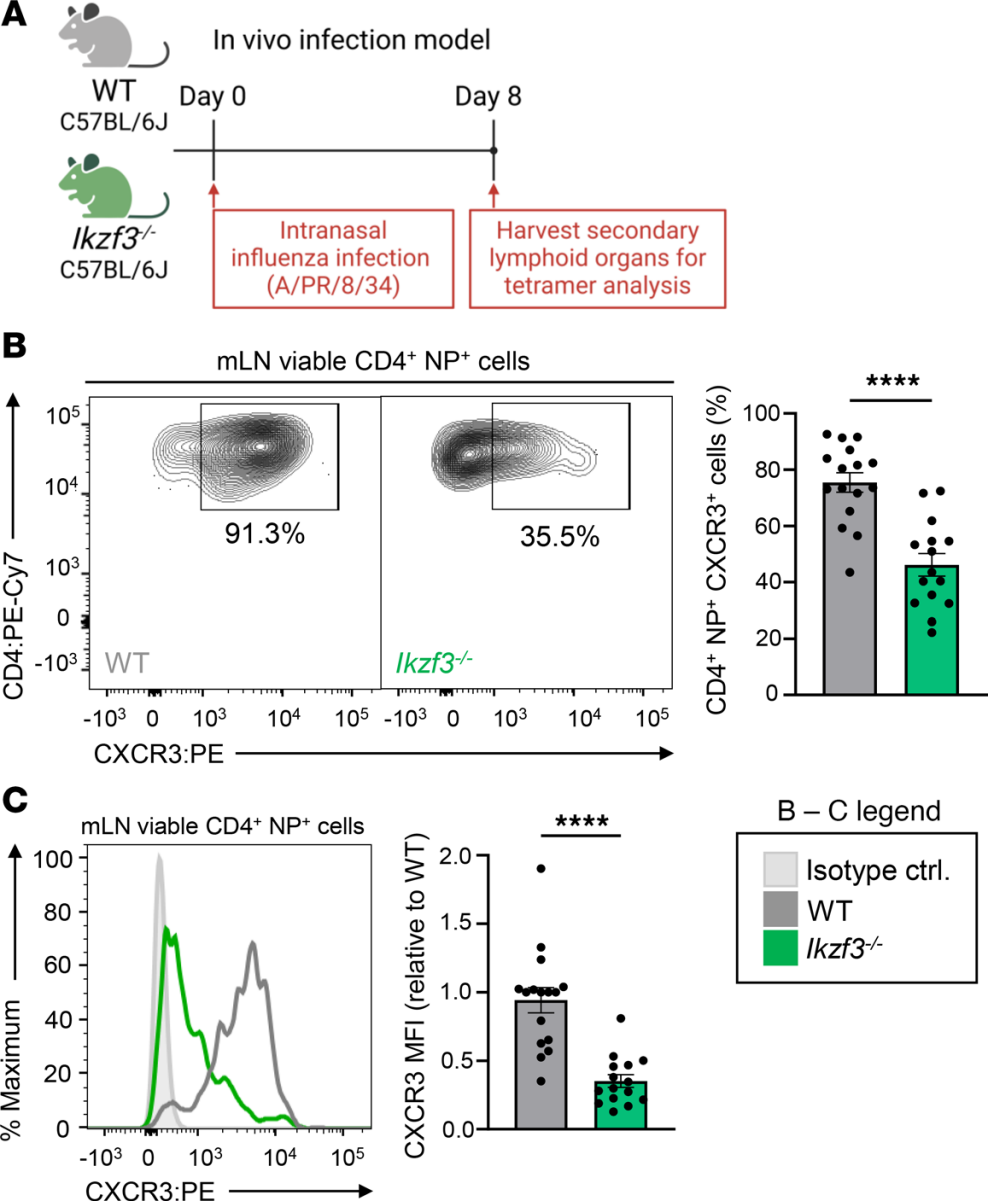
CXCR3 expression is reduced on Aiolos-deficient CD4^+^ T cells responding to IAV infection. (**A**) Schematic of murine model of IAV infection. WT or *Ikzf3^–/–^* mice were infected intranasally with 30 PFU of IAV (A/PR/8/34; PR8). After 8 days, mediastinal lymph nodes (mLN) and lungs were harvested and stained for flow cytometric analysis. Fluorochrome-labeled MHC class II tetramers were used to identify IAV nucleoprotein-specific (NP-specific) CD4^+^ T cells. (**B**) Representative flow cytometric analysis for CXCR3 expression in NP-specific CD4^+^ T cells isolated from the mLNs of WT or *Ikzf3^–/–^* mice. Data are compiled from 4 independent experiments and displayed as percentage positive for CXCR3. (**C**) Representative histogram overlay for CXCR3. Data are displayed as MFI fold-change compared with WT controls (*n* = 16 for WT and *n* = 15 for *Ikzf3^–/–^*, mean ± SEM; ****P < 0.0001, 2-tailed unpaired Student’s *t* test).

We next examined the impact of Aiolos deficiency on CXCR3 expression in vivo using a murine model of influenza A virus (IAV) infection (45). WT and *Ikzf3^–/–^* mice were intranasally infected with a sublethal dose of IAV strain A/PR/8/34 (H1N1, termed PR8), and nucleoprotein-specific (NP-specific) CD4^+^ T cells of the draining mediastinal lymph node (mLN) and lungs were assessed at 8 days after infection (Figure 2A). There was no significant difference in the numbers of NP-specific CD4^+^ T cells in the mLN between WT and *Ikzf3^–/–^* mice (Supplemental Figure 1A; supplemental material available online with this article; https://doi.org/10.1172/jci.insight.180287DS1). Further analyses of the NP-specific population revealed a significant decrease in CXCR3 surface expression in the absence of Aiolos (Figure 2, B and C). In contrast with the mLN, *Ikzf3^–/–^* mice had a significant reduction in NP-specific CD4^+^ T cells in the lungs compared with WT (Supplemental Figure 1B). These findings are in agreement with previous reports identifying CXCR3 as an essential chemokine receptor for antigen-specific effector T cell recruitment to the lungs (46, 47).

The numbers of bulk CD4^+^ T cells in the mLN were quantified and again revealed no significant difference between WT and *Ikzf3^–/–^* mice (Supplemental Figure 1C). However, in the lungs, we observed a significant reduction in the number of bulk CD4^+^ T cells in *Ikzf3^–/–^* mice compared with WT, though not to the extent observed with NP-specific cells (Supplemental Figure 1D). Collectively, these data suggest that migration of CD4^+^ T cells is disrupted in Aiolos-deficient mice during pulmonary infection.

T-bet is a known positive regulator of CXCR3 expression (48–51). Thus, we next examined T-bet expression to determine whether differences in this transcriptional regulator may explain the decrease in CXCR3 expression. However, we observed no significant difference in T-bet expression between NP-specific WT and *Ikzf3^–/–^* cells, suggesting that Aiolos-dependent regulation of CXCR3 expression may occur through a T-bet–independent mechanism (Supplemental Figure 2A).

Finally, we examined CXCR3 surface expression on bulk CD4^+^ naive, central memory, and effector T cell populations from the spleen to determine whether the decrease in CXCR3 expression was limited to distinct CD4^+^ T cell subsets. As expected, naive CD4^+^ T cells from both WT and *Ikzf3^–/–^* mice did not express CXCR3 (Supplemental Figure 2B). However, both central memory and effector CD4^+^ T cells from *Ikzf3^–/–^* mice displayed significantly reduced CXCR3 expression compared with their WT counterparts, with a greater reduction present on effector CD4^+^ T cells (Supplemental Figure 2, C and D). Overall, our findings in NP-specific and bulk CD4^+^ T cells suggest that Aiolos regulates CXCR3 expression in vivo.

### CXCR3 expression is reduced on Aiolos-deficient CD4^+^ T cells in a cell-intrinsic manner.

**Figure 3:**
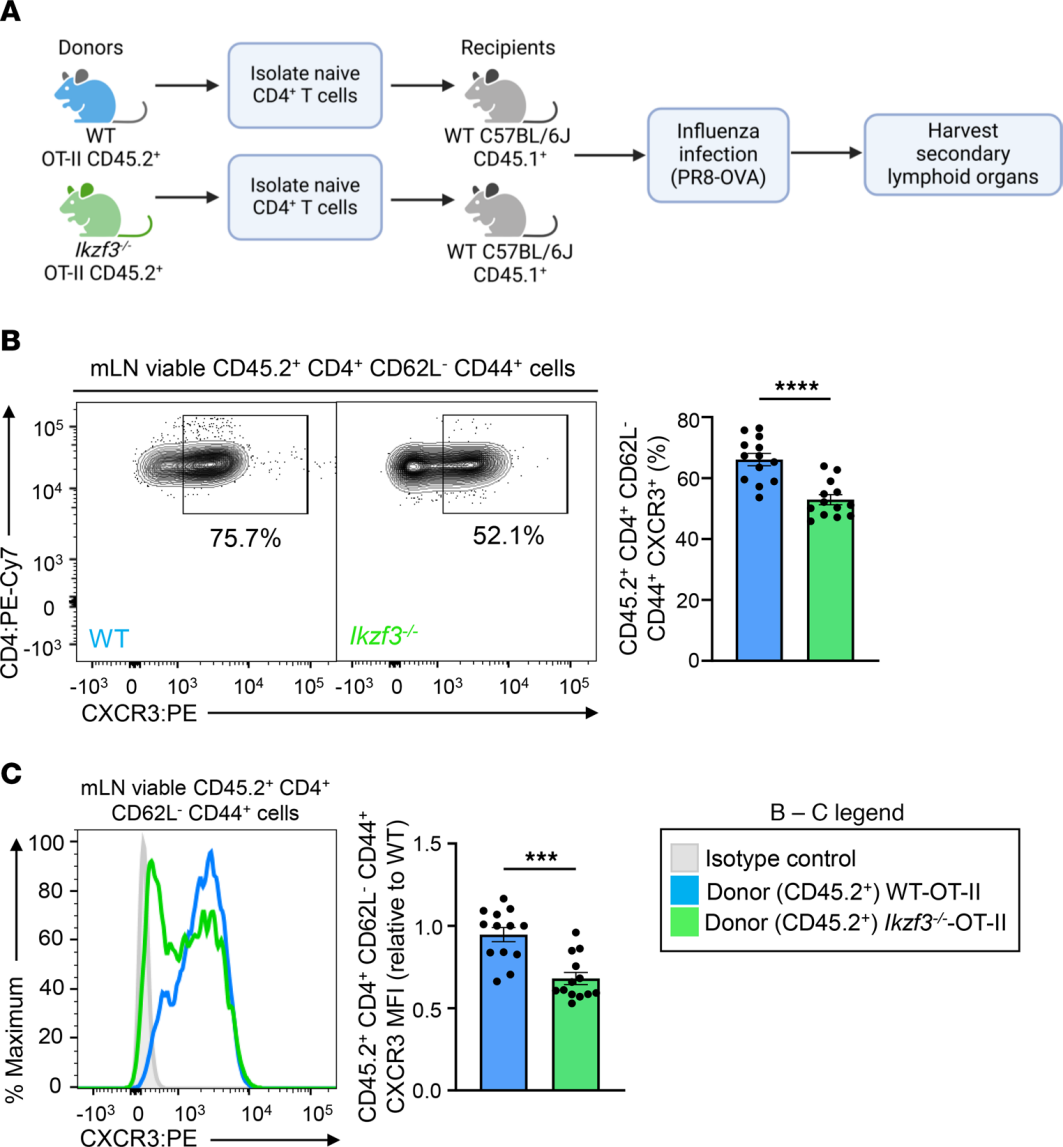
CXCR3 expression is reduced on Aiolos-deficient CD4^+^ T cells in a cell-intrinsic manner. (**A**) Schematic of adoptive transfer system. Naive CD4^+^ T cells were harvested from the mLNs of WT OT-II or *Ikzf3^–/–^* OT-II mice. A total of 500,000 cells/animal were adoptively transferred into CD45.1^+^ recipients. Recipient mice were then infected with 40 PFU of OVA_323–339_–expressing A/PR/8/34 (PR8-OVA) influenza virus 24 hours after transfer. At 8 days after infection, mLNs were harvested, and viable CD45.2^+^CD4^+^CD62L^–^CD44^+^ (antigen-specific, donor effector) cells were analyzed via flow cytometry. (**B**) Representative flow cytometric analysis for CXCR3 expression in CD45.2^+^CD4^+^CD62L^–^CD44^+^ cells in the mLN. Data are compiled from 3 independent experiments and displayed as percentage positive for CXCR3. (**C**) Representative histogram overlay for CXCR3. Data are displayed as MFI fold-change compared with WT OT-II control cells (*n* = 13, mean ± SEM; ***P < 0.001, ****P < 0.0001, 2-tailed unpaired Student’s *t* test).

To assess whether the impact of Aiolos on CXCR3 expression was CD4^+^ T cell intrinsic, we crossed WT and *Ikzf3^–/–^* mice onto the OT-II background, which expresses a transgenic TCR specific for the ovalbumin 323–339 peptide (OVA). We then adoptively transferred naive CD45.2^+^ CD4^+^ T cells from either WT OT-II or *Ikzf3^–/–^* OT-II mice into WT CD45.1^+^ recipients. Recipient mice were subsequently infected with OVA_323–339_–expressing PR8 (PR8-OVA) 24 hours after transfer, and antigen-specific CD45.2^+^ donor cells from the mLN were analyzed via flow cytometry at 8 days after infection (Figure 3A). Consistent with our findings in Aiolos-deficient mice, we observed a significant decrease in CXCR3 expression on donor CD45.2^+^ *Ikzf3^–/–^* cells compared with WT (Figure 3, B and C). We also noted a slight downregulation in T-bet expression in *Ikzf3^–/–^* compared with WT CD45.2^+^ donor cells (Supplemental Figure 3A). However, the fold-reduction in CXCR3 expression was greater than that of T-bet. Collectively, these findings demonstrate that the impact of Aiolos on CXCR3 expression occurs in a CD4^+^ T cell–intrinsic manner.

We next quantified the numbers of antigen-specific CD45.2^+^ donor cells in the mLN and lungs of recipient mice. In the mLN, there was no significant difference between the number of WT OT-II and *Ikzf3^–/–^* OT-II cells and only a slight but significant decrease in the frequency of Aiolos-deficient cells (Supplemental Figure 3B). In contrast with our findings in germline knockout animals, there was no significant difference in cell numbers or percentages between donor WT OT-II and *Ikzf3^–/–^* OT-II cells in the lungs (Supplemental Figure 3C). These data indicate that the migration of donor T cells to the lungs is not impaired when cells are intravenously injected into recipient mice, despite the observed reduction in CXCR3 expression. Thus, while Aiolos regulates CXCR3 expression in CD4^+^ T cell–intrinsic manner, alterations in CXCR3 expression do not ultimately have a cell-intrinsic effect on migration of adoptively transferred cells to the lungs.

To determine a potential explanation, we analyzed published RNA-Seq data (GSE203065) from WT and *Ikzf3^–/–^* Th1 cells. As with *Cxcr3*, various adhesion molecules and integrin subunits were downregulated in the absence of Aiolos. However, several other chemokine receptors known to promote Th1 cell migration (i.e., *Ccr5*, *Cxcr6*) were upregulated in Aiolos-deficient cells relative to WT (Supplemental Figure 3D). Hence, the impact of Aiolos on migratory programming appears to be multilayered with alterations to other migratory receptors potentially compensating for the loss of CXCR3 in the adoptive transfer setting.

### Aiolos deficiency alters expression of components of the IFN-γ/STAT1 and IL-12/STAT4 pathways.

**Figure 4:**
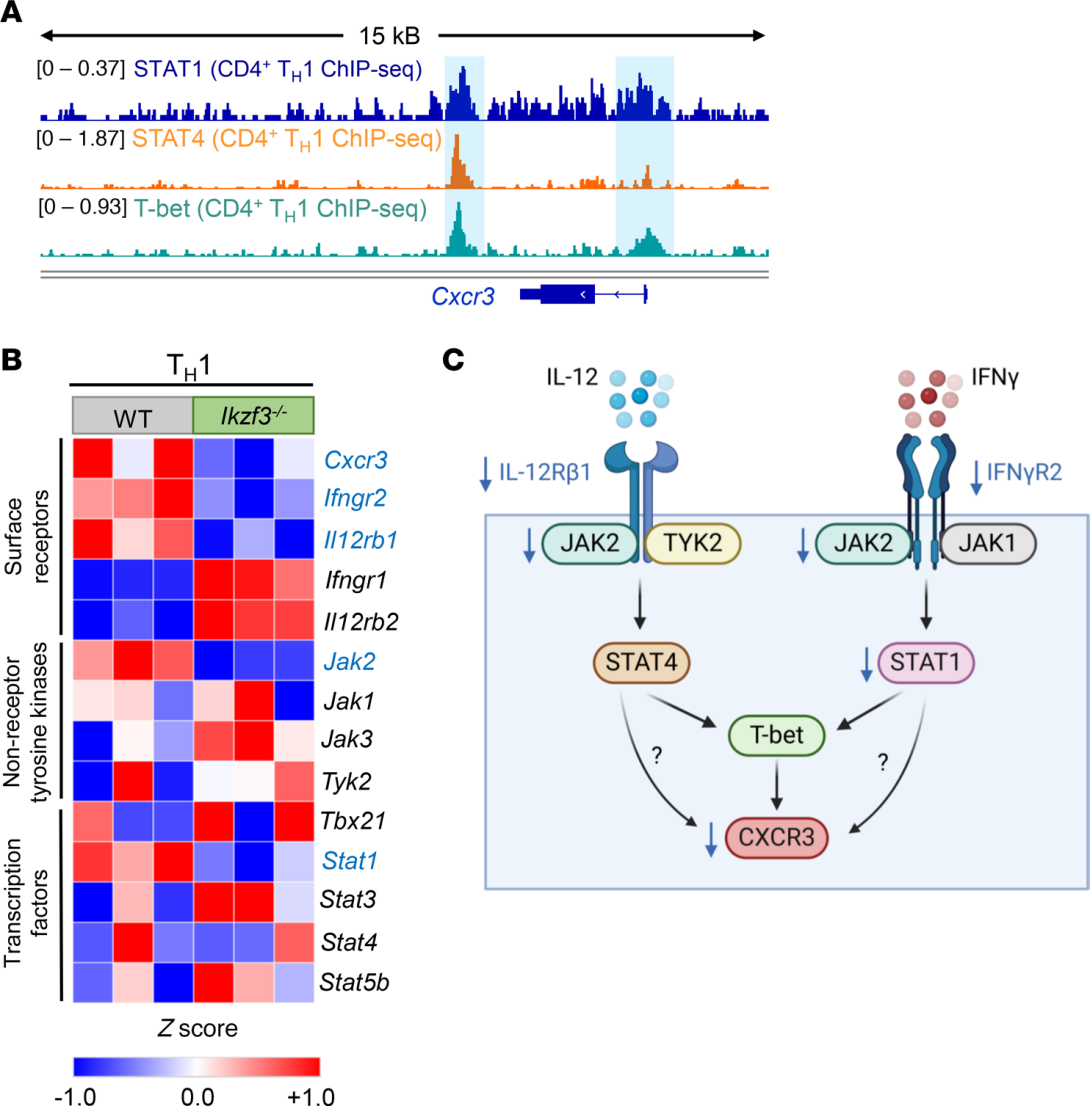
Aiolos-deficient Th1 cells exhibit altered expression of components of IFN-γ/STAT1 and IL-12/STAT4 signaling pathways. (**A**) Publicly available chromatin immunoprecipitation sequencing (ChIP-Seq) data for STAT1 (GSM994528), STAT4 (GSM550303), and T-bet (GSM836124) were examined at *Cxcr3*. Sequencing tracks were viewed using the Integrative Genomics Viewer (IGV). Regulatory regions of interest with transcription factor enrichment are indicated by the blue boxes. (**B**) Published RNA-Seq data (GSE203065) from in vitro–generated WT and *Ikzf3^–/–^* Th1 cells were analyzed for DEGs. A heatmap of DEGs associated with IFN-γ/STAT1 and IL-12/STAT4 signaling in Th1 cells is shown, as well as additional genes involved in both pathways and Th cell differentiation. Gene names color-coded in blue are downregulated in *Ikzf3^–/–^* Th1 cells. Note: *Cxcr3* transcript data presented here are the same as in Supplemental Figure 3D. (**C**) Schematic of proposed model in which Aiolos may regulate CXCR3 via impacts on components of the IFN-γ/STAT1 and IL-12/STAT4 cytokine signaling pathways. The downward arrows in blue indicate genes that are downregulated in the absence of Aiolos.

We next sought to identify the mechanism(s) by which Aiolos may regulate CXCR3 expression in Th1 cells. In addition to T-bet, CXCR3 expression is regulated by STAT1 and STAT4 (44, 52–54). Comparison of publicly available chromatin immunoprecipitation sequencing (ChIP-Seq) data for STAT1 (ChIP Atlas GSM994528), STAT4 (GSM550303), and T-bet (GSM836124) revealed enrichment of these factors at the promoter and 3′ enhancer regions of the *Cxcr3* locus (Figure 4A) (48–51, 55–58). Analysis of published RNA-Seq data (National Center for Biotechnology Information Gene Expression Omnibus GSE203065) from WT and *Ikzf3^–/–^* Th1 cells showed that the expression of key components of the IFN-γ/STAT1 and IL-12/STAT4 signaling pathways was altered in the absence of Aiolos. Specifically, *Jak2* and *Stat1* were downregulated in *Ikzf3^–/–^* compared with WT Th1 cells (Figure 4B) (28). Notably, *Jak2*, which is shared between the IFN-γ and IL-12 pathways, was the only JAK that was significantly downregulated in *Ikzf3^–/–^* Th1 cells (findings available in Supporting Data Values). Further, genes encoding the IFN-γ and IL-12 cytokine receptor subunits, *Ifngr2* and *Il12rb1*, also displayed slight decreases in the absence of Aiolos (Figure 4B). Given that many of the altered genes encode proteins involved in IFN-γ/STAT1 and IL-12/STAT4 signaling, we hypothesized that Aiolos may regulate CXCR3 via impacts on these pathways (Figure 4C).

### IFN-γ/STAT1 signaling, but not IL-12/STAT4, is diminished in the absence of Aiolos.

**Figure 5:**
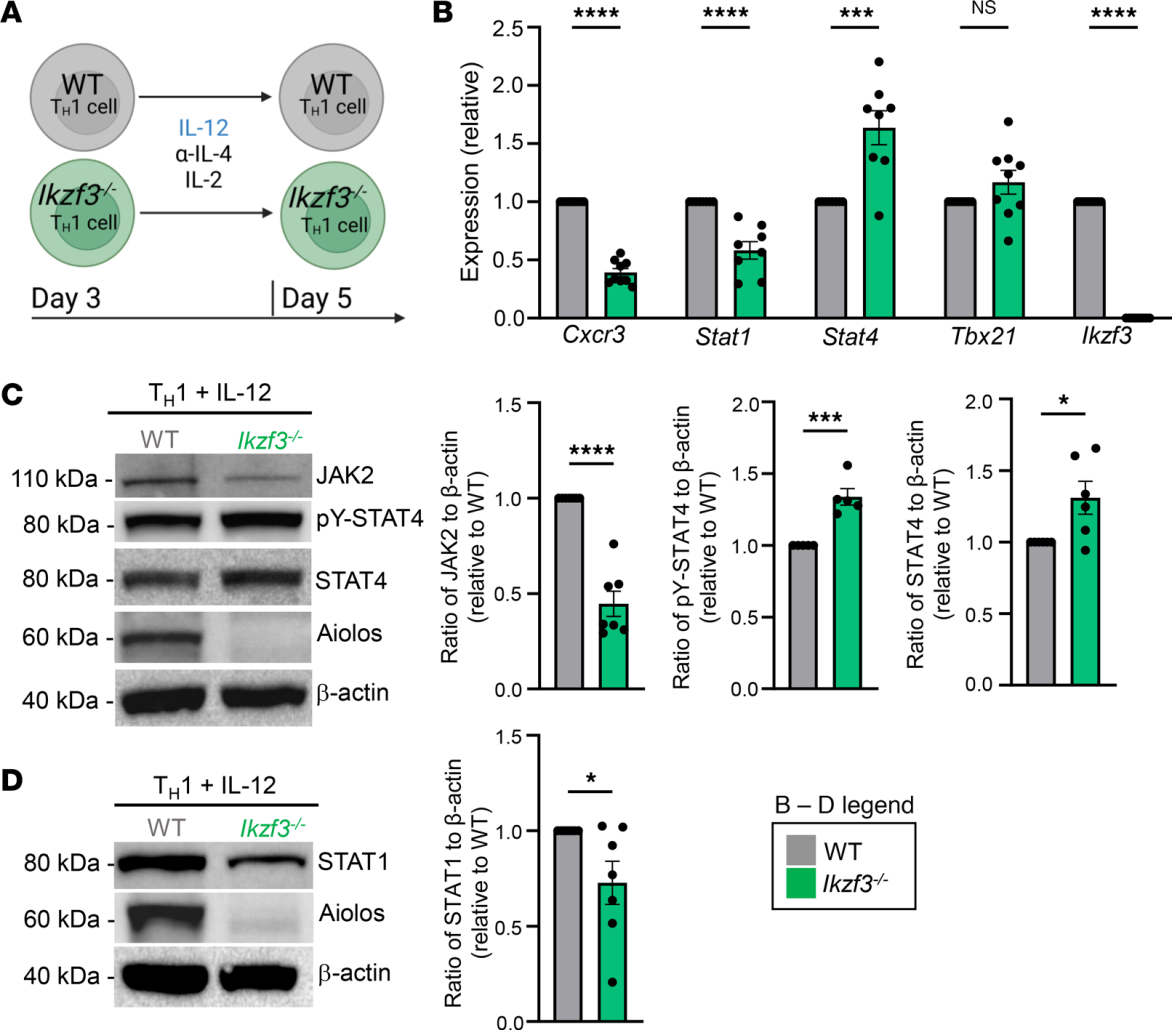
IFN-γ/STAT1 signaling, but not IL-12/STAT4, is diminished in IL-12–treated, Aiolos-deficient Th1 cells. (**A**) Schematic of culturing system. Naive CD4^+^ T cells were stimulated with α-CD3/CD28 under Th1-polarizing conditions (IL-12, α–IL-4). On day 3, cells were removed from stimulation and placed back into fresh Th1-polarizing conditions (IL-12, α–IL-4) with IL-2 for an additional 2 days. (**B**) At day 5, transcript analysis was performed via qRT-PCR. Transcript was normalized to *Rps18* and presented as fold-change compared with WT control (*n* = 8–9 biological replicates from 8–9 independent experiments. Data are presented as mean ± SEM; ***P < 0.001, ****P < 0.0001, 2-tailed unpaired Student’s *t* test). Note: *Cxcr3* and *Ikzf3* transcript data presented here are the same as in Figure 1E. (**C** and **D**) An immunoblot was performed to assess the relative abundance of the indicated proteins. β-Actin serves as a loading control (*n* = 5–7 independent experiments, mean ± SEM; *P < 0.05, ***P < 0.001, ****P < 0.0001, 2-tailed unpaired Student’s *t* test).

We next assessed the impact(s) of Aiolos on IFN-γ/STAT1 and IL-12/STAT4 signaling with WT and *Ikzf3^–/–^* CD4^+^ T cells cultured under Th1 conditions. Previous work from our lab has established that Aiolos-deficient Th1 cells have enhanced IFN-γ production upon stimulation (28). To control for this, as well as any other cytokines produced by CD4^+^ T cells in culture, cells were removed from stimulation on day 3 of differentiation and cultured for an additional 2 days with IL-12 before being harvested for analysis (Figure 5A). Transcript analysis of IL-12–treated cells revealed significant decreases in *Cxcr3*, *Stat1*, *Jak2*, and *Ifngr2* in *Ikzf3^–/–^* relative to WT Th1 cells. In contrast, transcript levels for *Stat4* and *Il12rb2* were significantly increased in *Ikzf3^–/–^* Th1 cells compared with WT (Figure 5B and Supplemental Figure 4A). No significant difference in transcript for *Tbx21* was observed between groups (Figure 5B). Immunoblot analyses similarly revealed significant reductions in total JAK2 and STAT1 protein in the absence of Aiolos (Figure 5, C and D). In contrast, tyrosine-phosphorylated STAT4 (pY-STAT4) and total STAT4 protein levels were significantly elevated in the absence of Aiolos (Figure 5C), demonstrating that the STAT4 pathway was not functionally inhibited by Aiolos deficiency when cells were treated with IL-12.

**Figure 6:**
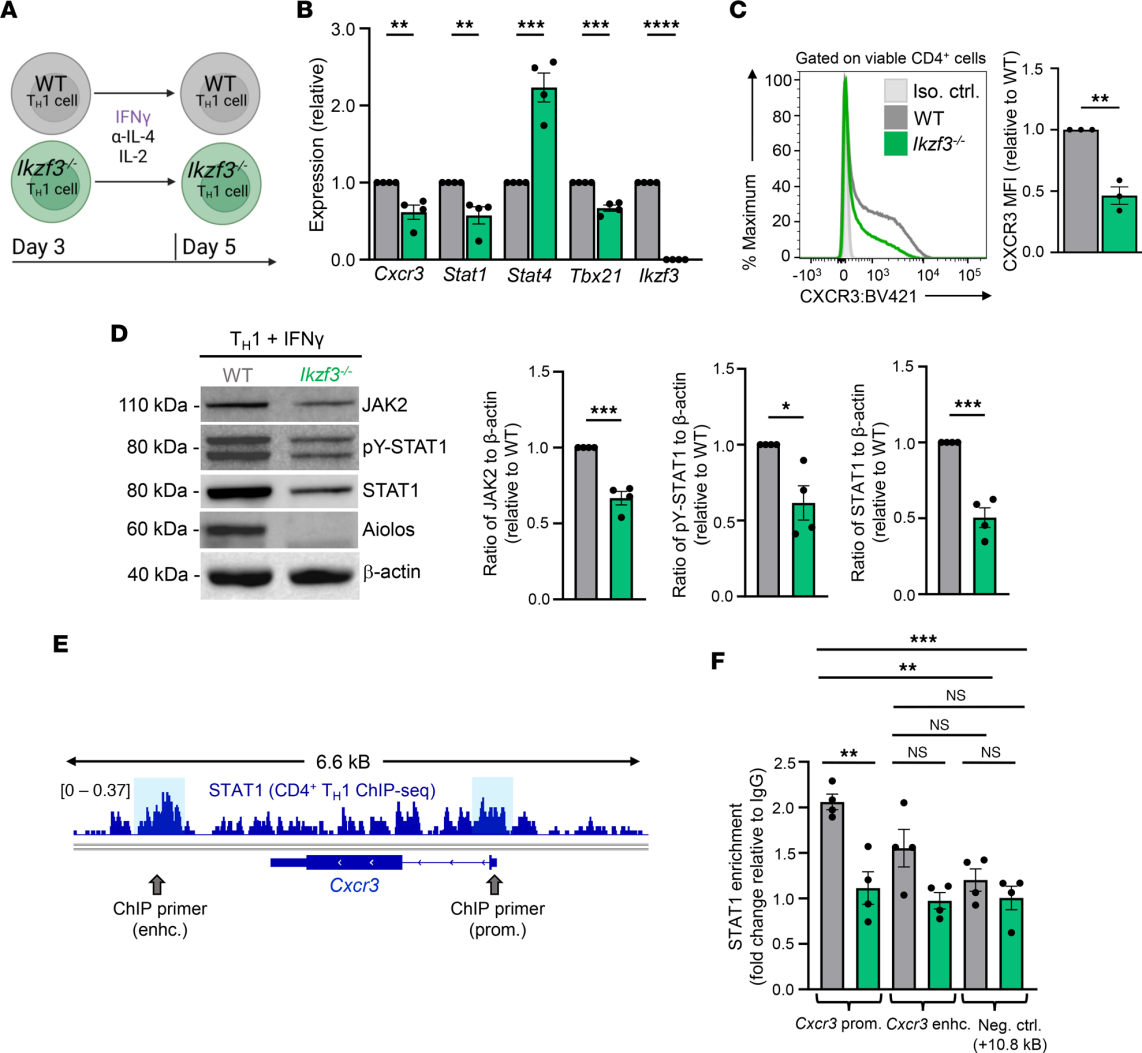
IFN-γ/STAT1 signaling is compromised in IFN-γ–treated, Aiolos-deficient Th1 cells. (**A**) Schematic of culturing system. Naive CD4^+^ T cells were stimulated with α-CD3/CD28 and cultured under Th1-polarizing conditions (IL-12, α–IL-4). On day 3, cells were removed from stimulation and given IFN-γ, α–IL-4, and IL-2 for an additional 2 days. (**B**) At day 5, transcript analysis was performed via qRT-PCR. Transcript was normalized to *Rps18* and presented as fold-change compared with WT control (*n* = 4 biological replicates from 4 independent experiments, mean ± SEM; **P < 0.01, ***P < 0.001, ****P < 0.0001, 2-tailed unpaired Student’s *t* test). (**C**) Representative flow cytometric analysis for CXCR3 on IFN-γ–treated Th1 cells at day 5. Data are displayed as MFI fold-change compared with WT controls (*n* = 3 biological replicates from 3 independent experiments, mean ± SEM; **P < 0.01, 2-tailed unpaired Student’s *t* test). (**D**) An immunoblot was performed to assess the relative abundance of the indicated proteins. β-Actin serves as a loading control (*n* = 4 independent experiments, mean ± SEM; *P < 0.05, ***P < 0.001, 2-tailed unpaired Student’s *t* test). (**E**) ChIP assays were performed to assess STAT1 association with *Cxcr3* in WT and *Ikzf3^–/–^* Th1 cells. Publicly available ChIP-Seq data for STAT1 (GSM994528) were examined to identify potential regions of STAT1 enrichment. Sequencing tracks were viewed using IGV, and regulatory regions of interest are indicated by blue boxes. Approximate ChIP primer locations at the *Cxcr3* promoter (prom.) and 3′ enhancer (enhc.) are indicated with gray arrows. (**F**) The indicated regions were analyzed for STAT1 enrichment. Data were normalized to total input. Percentage enrichment relative to input was divided by IgG, and data are presented as fold-change relative to IgG. (*n* = 4 biological replicates from 4 independent experiments, mean ± SEM; **P < 0.01, ***P < 0.001, 1-way ANOVA with Tukey’s multiple comparisons test.)

To more directly assess the impact of Aiolos deficiency on the IFN-γ/STAT1 signaling pathway, we cultured WT and *Ikzf3^–/–^* CD4^+^ T cells under Th1 conditions for 3 days, then added IFN-γ, rather than IL-12, for an additional 48 hours in the absence of stimulation (Figure 6A). Again, *Ikzf3^–/–^* Th1 cells had significantly reduced expression of *Cxcr3*, *Stat1*, *Jak2*, and *Ifngr2* compared with WT, whereas *Stat4* and *Il12rb2* transcripts were consistently increased (Figure 6B and Supplemental Figure 4B). In contrast with IL-12–treated cells, we also found that *Tbx21* expression was significantly decreased in Aiolos-deficient cells, suggesting that IFN-γ/STAT1 signaling is unable to compensate for the lack of IL-12/STAT4–dependent activation of T-bet expression in the absence of Aiolos (Figure 6B) (54).

Flow cytometry analysis similarly revealed a significant decrease in CXCR3 surface expression on *Ikzf3^–/–^* cells compared with WT (Figure 6C). Immunoblot analyses showed significant reductions in total JAK2, pY-STAT1, and total STAT1 protein in the absence of Aiolos (Figure 6D). However, total STAT4 protein remained significantly elevated in *Ikzf3^–/–^* Th1 cells (Supplemental Figure 4C). Thus, across IL-12 and IFN-γ stimulation conditions, only JAK2 and STAT1 correlated with the reduced CXCR3 expression observed in the absence of Aiolos.

We next examined publicly available STAT1 ChIP-Seq data (GSM994528) to identify STAT1-binding regulatory regions at the *Cxcr3* locus that may be affected by Aiolos deficiency and performed ChIP analysis of IFN-γ–treated WT versus *Ikzf3^–/–^* Th1 cells (Figure 6E) (56). Coincident with the loss of STAT1 expression, we observed a significant decrease in STAT1 enrichment at the *Cxcr3* promoter and a trending decrease in STAT1 enrichment at the *Cxcr3* 3′ enhancer region (*P* = 0.0697) in the absence of Aiolos (Figure 6F). These collective data demonstrate that the IFN-γ/STAT1 pathway is compromised by Aiolos deficiency and that Aiolos functions to positively regulate CXCR3 expression via STAT1.

### IFN-γ/STAT1 signaling induces Aiolos expression.

**Figure 7:**
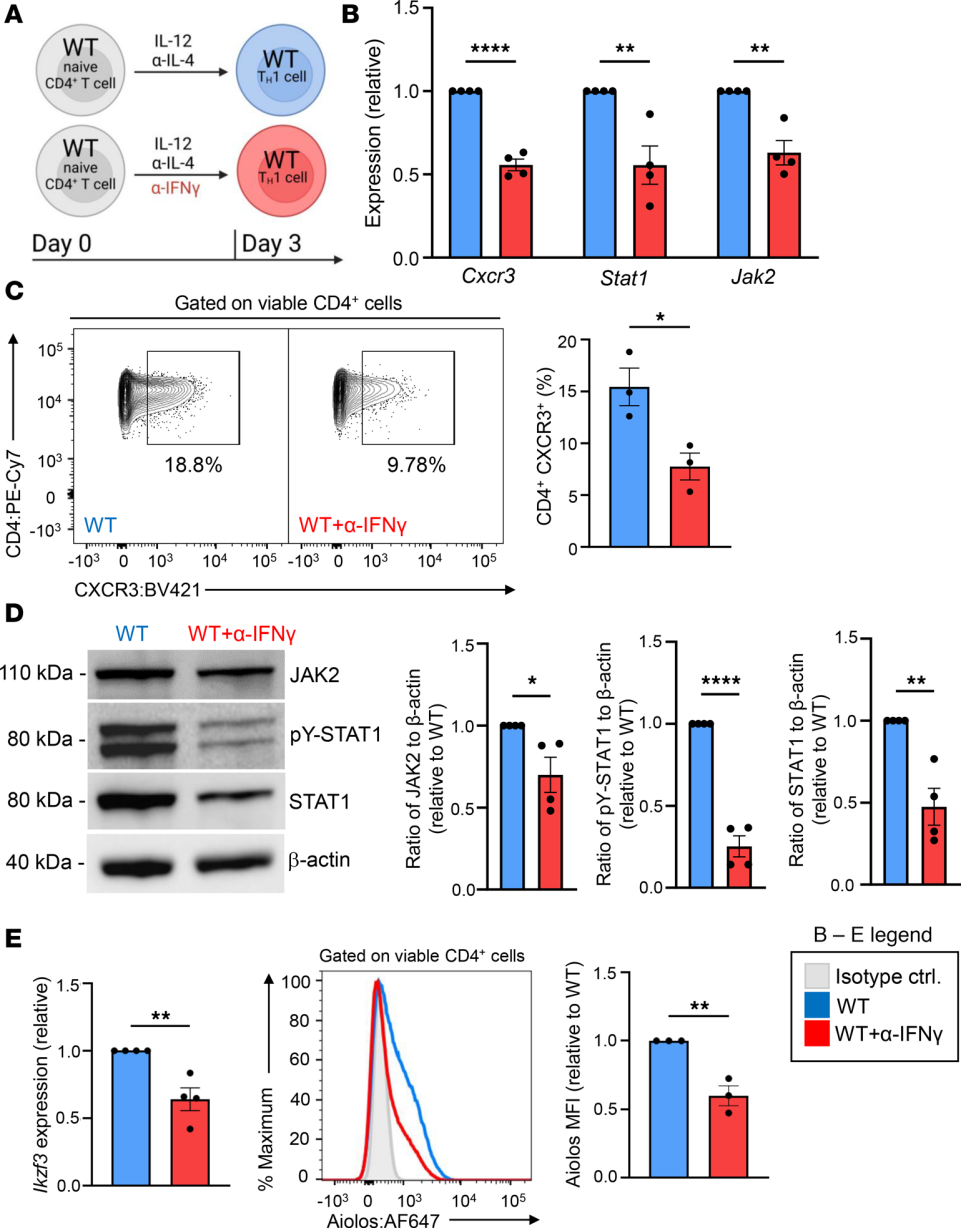
IFN-γ/STAT1 signaling induces Aiolos expression. (**A**) Schematic of culturing system. WT naive CD4^+^ T cells were stimulated with α-CD3/CD28 under Th1-polarizing conditions (IL-12, α–IL-4). Some cells were also treated with α–IFN-γ to inhibit IFN-γ/STAT1 signaling. (**B**) At day 3, transcript analysis was performed via qRT-PCR. Transcript was normalized to *Rps18* and presented as fold-change compared with WT control (*n* = 4 biological replicates from 4 independent experiments, mean ± SEM; **P < 0.01, ****P < 0.0001, 2-tailed unpaired Student’s *t* test). (**C**) Representative flow cytometric analysis at day 3 for CXCR3 expression on WT Th1 cells treated with or without α–IFN-γ. Data are displayed as percentage positive for CXCR3 (*n* = 3 biological replicates from 3 independent experiments, mean ± SEM; *P < 0.05, 2-tailed unpaired Student’s *t* test). (**D**) An immunoblot was performed to assess the relative abundance of the indicated proteins. β-Actin serves as a loading control (*n* = 4 independent experiments, mean ± SEM; *P < 0.05, **P < 0.01, ****P < 0.0001, 2-tailed unpaired Student’s *t* test). (**E**) At day 3, transcript and flow cytometric analyses were performed for *Ikzf3* and Aiolos protein expression, respectively. Flow cytometric data are displayed as MFI fold-change compared with WT controls (*n* = 3 biological replicates from 3 independent experiments, mean ± SEM; **P < 0.01, 2-tailed unpaired Student’s *t* test).

We next wanted to examine how directly inhibiting IFN-γ signaling may affect the relationship between Aiolos, STAT1, and CXCR3. We cultured WT naive CD4^+^ T cells under Th1 conditions for 3 days with the addition of an IFN-γ–neutralizing antibody to inhibit autocrine IFN-γ signals (Figure 7A). Neutralizing IFN-γ led to significant reductions in *Cxcr3*, *Stat1*, and *Jak2* expression (Figure 7B) (44, 52). Flow cytometric and immunoblot analyses similarly revealed significant reductions in CXCR3, total JAK2, total STAT1, and pY-STAT1 protein levels when IFN-γ was neutralized (Figure 7, C and D). Notably, Aiolos expression was also significantly decreased with IFN-γ neutralization, suggesting a possible positive feedback loop between Aiolos and IFN-γ/STAT1 (Figure 7E).

### Aiolos and STAT1 are enriched at the Stat1 and Ikzf3 promoters, respectively.

**Figure 8:**
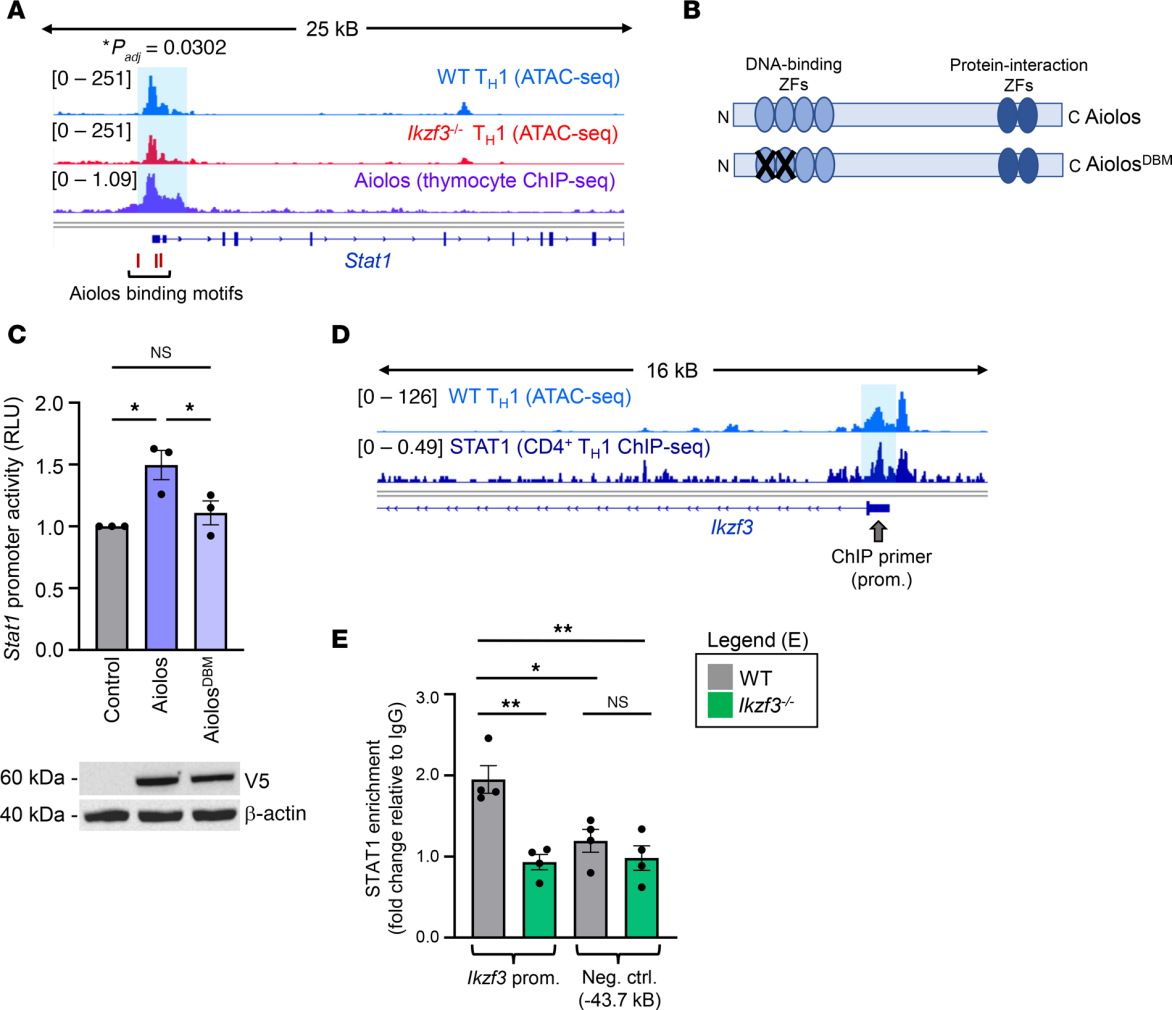
Aiolos and STAT1 engage in reciprocal regulation. (**A**) Publicly available ATAC-Seq data (GSE203064) from WT and *Ikzf3^–/–^* Th1 cells were assessed for alterations in chromatin accessibility at the *Stat1* promoter. Publicly available ChIP-Seq data for Aiolos (GSM5106065) were examined at *Stat1*. Sequencing tracks were viewed using IGV. The *Stat1* promoter region of significant differential accessibility is indicated by a blue box (*P_adj_* = 0.0302). A ~500 bp region encompassing the indicated Aiolos DNA binding motifs within the *Stat1* promoter was subcloned into a reporter plasmid. (**B**) Schematic depicting the zinc finger (ZF) domains of WT Aiolos and a DNA-binding mutant (Aiolos^DBM^). (**C**) EL4 T cells were transfected with a *Stat1* promoter-reporter and WT Aiolos, Aiolos^DBM^, or empty vector control. Cells were also transfected with SV40-*Renilla* as a control for transduction efficiency. Luciferase promoter-reporter values were normalized to *Renilla* control and presented relative to the empty vector control. Aiolos was assessed via immunoblot with an antibody for the V5 epitope tag. β-Actin serves as a loading control. Data are representative of 3 independent experiments (*n* = 3, mean ± SEM; *P < 0.05, 1-way ANOVA with Tukey’s multiple comparisons test). (**D**) Publicly available ATAC-Seq data (GSE203064) from Th1 cells and ChIP-Seq data for STAT1 (GSM994528) were viewed using IGV to identify regions of STAT1 enrichment (blue box) at *Ikzf3*. Approximate ChIP primer locations are indicated with a gray arrow. (**E**) The *Ikzf3* promoter (prom.) and a negative control region (neg. ctrl.) were analyzed for STAT1 enrichment via ChIP. Data were normalized to total input. Percentage enrichment relative to input was divided by IgG, and data are presented as fold-change relative to IgG. (*n* = 4 biological replicates from 4 independent experiments, mean ± SEM; *P < 0.05, **P < 0.01, 1-way ANOVA with Tukey’s multiple comparisons test.)

Given the positive correlation between Aiolos and STAT1 expression, we performed an in silico analysis for the core IkZF factor DNA-binding motif GGGAA at the *Stat1* locus and found several predicted binding sites at the promoter region. Subsequent examination of publicly available Aiolos ChIP-Seq data (GSM5106065) revealed a region of Aiolos enrichment at the *Stat1* promoter (Figure 8A) (23). These findings suggested that *Stat1* could be a direct target gene of Aiolos in Th1 cells. Since IkZF factors are known regulators of chromatin structure, we examined previously published assay for transposase-accessible chromatin sequencing (ATAC-Seq) data (GSE203064) from WT and *Ikzf3^–/–^* cells cultured under Th1 conditions. Indeed, in the absence of Aiolos, we observed a significant decrease in chromatin accessibility at the *Stat1* promoter (Figure 8A) (28).

To test whether Aiolos could regulate *Stat1* promoter activity, we created a *Stat1* promoter-reporter construct encompassing the Aiolos-enriched region. We then overexpressed with WT Aiolos or an Aiolos DNA-binding mutant (Aiolos^DBM^) containing point mutations in the first 2 N-terminal ZF domains, rendering this domain nonfunctional (Figure 8B) (18). We observed a significant increase in *Stat1* promoter activity upon overexpression of WT Aiolos, and this induction was lost upon overexpression of Aiolos^DBM^ (Figure 8C). These findings indicate that Aiolos is capable of inducing *Stat1* promoter activity and that the DNA-binding domain is required.

Last, to determine whether STAT1 may reciprocally regulate Aiolos expression, we examined publicly available ChIP-Seq data for STAT1 (GSM994528) and previously published ATAC-Seq data (GSE203064) from WT Th1 cells. Indeed, we identified a potential region of STAT1 enrichment at the *Ikzf3* promoter, which correlated with a region of accessible chromatin in Th1 cells (Figure 8D) (28, 56). Further, ChIP analysis of IFN-γ–treated WT Th1 cells revealed that STAT1 was enriched at the *Ikzf3* promoter relative to an upstream control region (Figure 8E). Collectively, these data support the existence of a positive feedback loop between Aiolos and STAT1 through which IFN-γ/STAT1 signaling and CXCR3 expression are regulated.

## Discussion

Aiolos has long been implicated in lymphoid cell development (16, 19–22). More recently, Aiolos has emerged as a regulator of cytokine signaling pathways and effector programs in innate and adaptive lymphocytes (17, 18, 27). Here, we have identified Aiolos as a positive regulator of IFN-γ/STAT1 signaling and a driver of CXCR3 expression in Th1 cells. Our observations are consistent with findings from earlier studies indicating that IFN-γ and STAT1 are critical for induction of CXCR3 on CD4^+^ T cells (44, 52). The work presented here expands upon those findings by uncovering Aiolos as a positive transcriptional regulator of STAT1 and that IFN-γ/STAT1 subsequently promotes Aiolos expression through a feed-forward amplification loop.

In contrast with STAT1, we found that expression of both tyrosine-phosphorylated and bulk STAT4 protein was elevated in Aiolos-deficient cells. Relatedly, others have reported an inverse correlation between activation of STAT4 and overall levels of STAT1 in NK cells (59). We also observed that Aiolos deficiency resulted in reduced expression of JAK2, a kinase of the IL-12/STAT4 signaling pathway. One explanation for this apparent contradiction may be that alterations in expression of other cytokine signaling pathway components offsets reduced JAK2 levels. For example, we have previously shown that Aiolos deficiency allows for enhanced IL-2 sensitivity, and it has been established that IL-2 induces expression of *Il12rb2* (28, 60). Previous work has demonstrated that TYK2, another nonreceptor tyrosine kinase involved in IL-12/STAT4 signaling, is critical for STAT4-mediated IFN-γ expression (61). Future studies are needed to determine whether TYK2 activation may contribute to sustained IL-12/STAT4 signaling despite the loss of JAK2 in the absence of Aiolos.

We found that adoptively transferred Aiolos-deficient cells do not exhibit altered migration to the lungs, despite reduced CXCR3 expression. In addition to CXCR3, the chemokine receptor CCR5 is also known to enable Th1 cell migration, and previous work has demonstrated that IL-12/STAT4 signaling selectively upregulates CCR5 (44, 62, 63). Indeed, we find elevated *Ccr5* transcript in Aiolos-deficient cells, suggesting that it may compensate for reduced CXCR3 expression. Finally, given that both STAT1 and STAT4 positively affect T-bet expression, the counter-regulatory nature of Aiolos on IFN-γ/STAT1 and IL-12/STAT4 signaling may help explain the inconsistent alterations in T-bet expression that we observed in the absence of Aiolos. Our finding that CXCR3 and T-bet expression do not consistently correlate further supports the conclusion that another Aiolos-dependent factor (i.e., STAT1) is responsible for regulating CXCR3 expression. Indeed, STAT1 has been shown to control Th1 trafficking to the lung through a T-bet–independent mechanism (64).

IFN-γ/STAT1 signaling operates across multiple cell types, suggesting that the regulatory mechanisms established here may extend to additional Aiolos-expressing immune cells, such as innate lymphoid cells, CD8^+^ T cells, and B cells (16, 65–73). It is also possible that Aiolos could affect other STAT1-driven cytokine pathways (i.e., IFN-α/β/λ, IL-27, IL-6) (74–76). For example, a recent study indicated that Aiolos promotes the proliferation and survival of HIV-1–infected cells, which was associated with the upregulation of genes involved in T cell migration and type I IFN responses (77). Work by others in CD8^+^ T cells has shown that reduced levels of STAT1, maintained by STAT4, are required for overcoming the antiproliferative effects of type I IFN during viral infection (59). This is just one example suggesting that Aiolos-dependent alterations in STAT1 expression could affect other signaling pathways.

Studies in humans have described *IKZF3* missense mutations that compromise the DNA-binding domain and result in primary immunodeficiency and inborn errors of immunity (23–26). Two such mutations, termed G159R and N160S, have been reported in humans and are associated with increased susceptibility to infections and dysregulated immune responses. The *IKZF3* G159R mutation has been associated with B cell deficiency, B cell malignancy, and abnormal T cell differentiation (23). Similarly, the *IKZF3* N160S mutation has been shown to affect both B and T cell populations, leading to increased susceptibility to infection. With regard to T cells, the *IKZF3* N160S mutation results in decreased Tfh, Th1, and memory T cell populations (25). Thus, our finding that Aiolos regulates IFN-γ/STAT1 signaling may provide at least some mechanistic explanation for these observed disruptions in humans. Overall, these findings underscore the clinical consequences arising from genetic defects in Aiolos function, notably including compromised Th1 responses.

While chemokine receptor expression is normally restricted to lymphocyte populations, many cancers are known to aberrantly express these receptors and consequently acquire migratory (metastatic) abilities, a phenomenon termed lymphocyte mimicry (78, 79). Aiolos has been linked to metastatic lung cancer through the induction of such pathways, including expression of CXCR4 (80–82). Of note, previous work from our lab has demonstrated that Aiolos promotes CXCR5 expression in Tfh cells, which enables their migration into B cell follicles (18, 28). More recent work has demonstrated that T and B cells homozygous for an Aiolos missense mutation exhibit impaired homing into lymph nodes primarily due to low CD62L expression (83). These studies, alongside our observation of disrupted CXCR3 expression and altered transcript levels for multiple cell adhesion molecules, integrin subunits, and chemokine receptors in Aiolos-deficient Th1 cells, suggest that Aiolos drives a larger immune cell migratory program, which ultimately requires further investigation.

Finally, JAK/STAT signaling pathways have long been the target of different therapeutics due to demonstrated roles in autoimmune diseases and hematological malignancies (6–9, 84–88). Similarly, Aiolos has been targeted therapeutically with lenalidomide, an immunomodulatory drug used to treat multiple myeloma and various lymphomas (89–92). Lenalidomide has also been shown to enhance the cytotoxic activity of CAR T cells against solid tumors, which is consistent with previous work showing that Aiolos suppresses T cell cytotoxic function (28, 93–95). However, given the current study, it is reasonable to postulate that loss of Aiolos could also result in altered lymphoid migratory patterns during immune responses to infection or cancer, which would be consistent with findings in human patients harboring Aiolos missense mutations (23–26). Hence, therapeutics that specifically target Aiolos may have disease-specific advantages and disadvantages, presenting a potential paradox. Ultimately, future studies will be required to determine the full extent of effects of Aiolos on immune cell programming, including its impact on STAT1-dependent signaling pathways.

## Methods

### Sex as a biological variable.

Germline knockout influenza virus infection studies and all in vitro experiments utilized both male and female mice to avoid unintentional sex bias. Similar findings are reported for both sexes. For adoptive transfer studies, only male donor and recipient mice were utilized due to the Y-linked nature of the OT-II transgene.

### Mouse strains.

WT CD45.1 (the Jackson Laboratory stock 002014) and CD45.2 C57BL/6J (the Jackson Laboratory stock 000664) mice were originally obtained from the Jackson Laboratory. Aiolos-deficient (*Ikzf3^–/–^*) mice were originally obtained from Riken BRC and were backcrossed onto the CD45.2 C57BL/6J Jackson Laboratory background for more than 10 generations. OT-II mice (the Jackson Laboratory stock 004194), with the transgene located on the Y chromosome, were originally generated by the Carbone laboratory (96) and were a gift from Haitao Wen (The Ohio State University, Columbus, Ohio, USA). For adoptive transfer studies, *Ikzf3^–/–^* mice were crossed to OT-II mice to generate *Ikzf3^–/–^* OT-II mice. For all experiments and replicates, individual mice were age and sex matched.

### CD4^+^ T cell isolation and culture.

Naive CD4^+^ T cells were isolated from the spleens and lymph nodes of 5- to 8-week-old mice using the BioLegend Mojo Sort naive CD4^+^ T cell isolation kit according to the manufacturer’s recommendations. For in vitro polarization of Th1 cell populations, naive CD4^+^ T cells were plated at a density of 300,000 cells/well in complete IMDM (IMDM [Life Technologies], 10% FBS [26140079, Life Technologies], 1% penicillin/streptomycin [Life Technologies], and 0.05% (50 μM) 2-mercaptoethanol [MilliporeSigma]). Plates were coated with αCD3 (clone 145-2C11; 5 μg/mL; BD Biosciences) and αCD28 (clone 37.51; 2 μg/mL; BD Biosciences) overnight and washed twice with PBS prior to the addition of cells in complete IMDM. Upon plating, cells were cultured in the presence of IL-4 neutralizing antibody (clone 11B11; 5 μg/mL; BioLegend) and the Th1-polarizing cytokine recombinant murine IL-12 (rmIL-12) (5 ng/mL; R&D Systems, Bio-Techne) for 72 hours prior to analysis or expansion. For experiments in which IFN-γ was neutralized, cells were also cultured in the presence of α–IFN-γ antibody (clone XMG1.2; 10 μg/mL; BioLegend) for 72 hours. For expansion of cells on day 3 into resting conditions, cells were plated at 500,000 cells/well in complete IMDM with the addition of fresh IL-4 neutralizing antibody (clone 11B11; 5 μg/mL; BioLegend), recombinant human IL-2 (250 U/mL; Peprotech), and either fresh rmIL-12 (5 ng/mL; R&D Systems, Bio-Techne) or rmIFN-γ (50 ng/mL; PeproTech), as noted. Cells were cultured for an additional 48 hours prior to harvesting for analysis on day 5. For experiments in which cells were cultured in the presence of rmIFN-γ, fresh rmIFN-γ (50 ng/mL; PeproTech) was added 1 hour prior to harvest.

### RNA isolation and qRT-PCR.

Total RNA was isolated from the cell populations described above using the MACHEREY-NAGEL Nucleospin RNA isolation kit according to the manufacturer’s guidelines. cDNA was generated using the Superscript IV First Strand Synthesis System (Thermo Fisher Scientific). qRT-PCRs were performed using the SYBR Select Mastermix for CFX (Thermo Fisher Scientific) with 10 ng cDNA per reaction and primers for the appropriate genes (Supplemental Table 1). All qRT-PCRs were performed on the CFX Connect (Bio-Rad). Data were normalized to *Rps18* and are presented as relative to the WT control sample.

### RNA-Seq analysis.

Published RNA-Seq data (GSE203065) from WT and *Ikzf3^–/–^* Th1 cells were analyzed as previously reported (28). Briefly, naive CD4^+^ T cells were cultured under Th1-polarizing conditions for 3 days. Total RNA was isolated using the MACHEREY-NAGEL Nucleospin RNA isolation kit according to the manufacturer’s guidelines. Samples were provided to Azenta Life Sciences for poly(A) selection, library preparation, sequencing, and DESeq2 analysis (3 biological replicates per genotype from 3 independent experiments). Genes with an adjusted *P* < 0.05 were considered differentially expressed. Heatmap generation and clustering (by Euclidean distance) were performed using normalized log_2_ counts from DESeq2 analysis and the Morpheus software (https://software.broadinstitute.org/morpheus/). Volcano plots were generated using –log_10_ (adjusted *P* value) and log_2_ fold-change values from DESeq2 analysis and VolcaNoseR software (https://huygens.science.uva.nl/VolcaNoseR/) (97).

### ATAC-Seq analysis.

Published ATAC-Seq data (GSE203064) from WT and *Ikzf3^–/–^* cells cultured under Th1 conditions were analyzed as previously described (28, 67). Briefly, 5 × 10^4^ cells with greater than 95% viability were processed with the Illumina Nextera DNA Library Preparation Kit according to the manufacturer’s instructions. Resultant sequences were trimmed and aligned to mm10 using Bowtie2. All subsequent analyses were performed using the indicated tools in Galaxy (https://usegalaxy.org). Samples were filtered by read quality (>30), as well as to remove duplicates and mitochondrial reads. Statistically significant peaks were identified using MACS2 callpeak. DiffBind (Bioconductor) was used to identify regions of significant differential accessibility between WT and *Ikzf3^–/–^* samples. Regions with adjusted *P* < 0.05 were considered statistically significant. Counts per million–normalized tracks were visualized using IGV versions 2.12.3 and 2.18.2.

### Immunoblot analysis.

Cells were harvested, counted, lysed in 1× SDS loading dye, (50 mM Tris at pH 6.8, 100 mM DTT, 2% SDS, 0.1% bromophenol blue, 10% glycerol) and boiled for 15 minutes. Equal protein lysate amounts were loaded based on cell counts. Lysates were separated via SDS-PAGE on 10% Bis-Tris Bolt gels (Thermo Fisher Scientific) and then transferred onto a 0.45 μm nitrocellulose membrane. Following transfer, nitrocellulose membranes were blocked with 2% nonfat dry milk in 1× TBST (10 mM Tris at pH 8.0, 150 mM NaCl, 0.05% Tween-20). The following antibodies were used to detect proteins: α-JAK2 (1:1,000; 3230, Cell Signaling Technology), α–pY-STAT4 (1:1,000; 5267, Cell Signaling Technology), α-STAT4 (1:1,000; 2653S, Cell Signaling Technology), α–pY-STAT1 (1:1,000; 9167S, Cell Signaling Technology), α-STAT1 (1:1,000; sc-417, Santa Cruz Biotechnology), α-Aiolos (1:20,000; 39293, Active Motif), α–β-actin–HRP (1:15,000; A00730, GenScript), goat α-mouse (1:5,000; 115-035-174, Jackson Immunoresearch), and mouse α-rabbit (1:5,000–1:10,000; sc-2357, Santa Cruz Biotechnology). Immunoblot bands were quantified using ImageJ (NIH) as previously described (27). For each protein row, the largest band was framed, and the mean gray value was measured using the same frame across the row. Background measurements were taken with the same frame measuring the area above or below bands in the image. Pixel densities were inverted, background values were subtracted from sample and control bands, and a ratio of net protein bands to net loading control bands was calculated for protein quantification relative to the WT sample.

### Influenza virus infection and tissue preparation.

IAV strain A/PR/8/34 (H1N1, PR8) and OVA_323–339_–expressing PR8 (PR8-OVA) were propagated in 10-day-old, specific pathogen–free embryonated chicken eggs (Charles River Laboratories) and titered on MDCK cells (BEI Resources, National Institute of Allergy and Infectious Diseases, NIH: Kidney [Canine], Working Cell Bank, catalog NR-2628). Mice between 8 and 12 weeks of age were infected intranasally with 30 plaque-forming units of PR8. After 8 days, mLNs, spleen, and lungs were harvested as previously described (28). For mLNs and spleen, single-cell suspensions were generated in tissue-processing media (IMDM + 4% FBS) by passing the tissue through a 100 μm nylon mesh strainer followed by erythrocyte lysis via a 3-minute incubation at room temperature in 0.84% NH_4_Cl. For lungs, single-cell suspensions were generated by incubating whole lung tissue in HBSS (Gibco) supplemented with 1.3 mM EDTA for 30 minutes at 37°C. Following this, lungs were processed in media (RPMI + 4% FBS) supplemented with Collagenase IV using a gentleMACS Dissociator (Miltenyi Biotec) for 30 minutes according to the manufacturer’s instructions. Samples were then filtered through a 40 μm nylon mesh strainer and subsequently centrifuged at 500*g* with a Percoll density gradient to isolate the mononuclear layer. Erythrocyte lysis was performed as previously described. For all tissues, cells were washed in FACS buffer (PBS + 4% FBS) prior to staining for flow cytometry. For adoptive transfer studies, naive CD45.2^+^ OT-II CD4^+^ T cells were purified from WT OT-II or *Ikzf3^–/–^* OT-II mice using negative selection as described above. Cells were washed and resuspended in sterile 1× PBS and transferred retro-orbitally (5 × 10^5^ cells/animal) into WT CD45.1^+^ recipient mice that were anesthetized with isoflurane. After 24 hours, mice were infected intranasally with 40 PFU of PR8-OVA.

### Flow cytometry.

For analysis of influenza NP-specific CD4^+^ T cells in germline *Ikzf3^–/–^* animals, cells were first incubated for at least 5 minutes at 4°C with TruStain FcX (anti-mouse CD16/32) Fc block (clone 93; 101320; BioLegend). Samples were then stained with IA^b^ NP_311–325_ MHC class II tetramer (1:100; NIH Tetramer Core Facility) in the presence of Fc block for 1 hour at room temperature protected from light. Extracellular markers were stained in the presence of Fc block for 30 minutes at 4°C protected from light using the following antibodies: αCD4 (PE/Cy7; 1:300; clone GK1.5; BD Biosciences, catalog 563933), αCD4 (APC; 1:300; clone GK1.5; BioLegend, catalog 100412), αCXCR3 (PE; 1:300; clone CXCR3-173; BioLegend, catalog 126505), αCXCR3 (BV421; 1:300; clone CXCR3-173; BioLegend, catalog 126529), αCD44 (V450; 1:300; clone IM7; BD Biosciences, catalog 560452), αCD44 (FITC; 1:300; clone IM7; Thermo Fisher Scientific, catalog 553133), αCD62L (APC-efluor780; 1:300; clone MEL-14; Thermo Fisher Scientific, catalog 47-0621-82), αCD45.1 (BV421; 1:300; clone A20; BioLegend, catalog 110732), and αCD45.2 (APC; 1:300; clone 104; BioLegend, catalog 109814). At the same time, cells were stained with Ghost viability dye (V510; 1:400; Tonbo Biosciences, catalog 13-0870-T100). Cells were then washed twice with FACS buffer prior to intracellular staining. For intracellular staining, cells were fixed and permeabilized using the eBioscience Foxp3 transcription factor staining kit (Thermo Fisher Scientific, catalog 00-5523-00) for 30 minutes or overnight at 4°C. After fixation, samples were stained with the following antibodies in 1× eBioscience permeabilization buffer (Thermo Fisher Scientific) for 30 minutes at room temperature protected from light: α–T-bet (PerCP-Cy5.5; 1:100; clone 4B10; BioLegend, catalog 644806) and α-Aiolos (AF647; 1:100; clone; S48-791; BD Biosciences, catalog 565265). Cells were washed twice with 1× permeabilization buffer and resuspended in FACS buffer for analysis. Samples were run on a BD Biosciences FACSCanto II flow cytometer and analyzed using FlowJo software (version 10.8.1, BD Biosciences). Representative gating strategies can be found in Supplemental Figures 5–7.

### Promoter-reporter assay.

A *Stat1* promoter-reporter construct (pGL3-*Stat1*) was generated by subcloning the regulatory region of *Stat1* (positions – 479 to 0 bp) into the pGL3-Promoter vector (Promega) (Supplemental Table 2). Aiolos contains 4 N-terminal ZF domains that mediate its DNA-binding capability. Of these 4 ZFs, ZF2 and ZF3 are required for DNA binding, whereas ZF1 and ZF4 are responsible for regulating sequence specificity (16–20). Expression vectors for WT Aiolos and an Aiolos^DBM^ were constructed as previously described (18). Briefly, 2 cystine residues in both ZF1 and ZF2 of Aiolos were mutated to alanine residues via site-directed mutagenesis, rendering the Aiolos DNA-binding domain nonfunctional. The EL4 murine T cell lymphoma line (TIB-39) was acquired from the American Type Culture Collection and maintained in complete RPMI (RPMI-1640, 10% FBS [26140079, Life Technologies], 1% penicillin/streptomycin [Life Technologies]). EL4 T cell transfections were performed using the Lonza 4D nucleofection system (program CM-120, buffer SF). EL4 cells were nucleofected with expression vectors for WT Aiolos, Aiolos^DBM^, or an empty vector control in conjunction with pGL3-*Stat1* and an SV40-*Renilla* vector as a control for transfection efficiency. After 22–24 hours of recovery, samples were harvested, and luciferase expression was analyzed using the Dual-Luciferase Reporter Assay System (Promega) according to the manufacturer’s instructions. Abundance of overexpressed proteins was assessed via immunoblot using an antibody against the V5 epitope tag (Thermo Fisher Scientific, catalog R960-25).

### ChIP.

ChIP assays were performed as described previously (98). In brief, chromatin was harvested from in vitro–generated Th1-like cells treated with IFN-γ. Chromatin was incubated with antibodies against STAT1 (Thermo Fisher Scientific, catalog 10144-2-AP; 7 μg per IP) or an IgG control (Abcam, catalog ab6709; 7 μg per IP, matched to experimental antibody), and the precipitated DNA was analyzed by qPCR with gene-specific primers (Supplemental Table 3). Samples were normalized to a total input DNA control, and percentage enrichment was divided by IgG values. The final value represents the percentage enrichment fold-change relative to the IgG control.

### Software summary.

Data were collected using the following open-source or commercially available software programs: BD Biosciences FACSDiva (version 8.0.2), Bio-Rad Image Lab (version 6.0.1, build 34), and Bio-Rad CFX Manager (version 3.1). Analyses and/or manuscript preparation were conducted using BD Biosciences FlowJo (version 10.8.1) and open-source software, including tools available on IGV (versions 2.12.3 and 2.18.2), Morpheus (https://software.broadinstitute.org/morpheus), VolcaNoseR (https://huygens.science.uva.nl/VolcaNoseR/), and Galaxy (https://usegalaxy.org). BioRender (https://biorender.com/) was used to create schematics and the graphical abstract under licenses (Supplemental Table 4). All statistical analyses were performed using GraphPad Prism software (version 10). Data preparation for this manuscript did not require the use of custom code or software.

### Statistics.

All statistical analyses were performed using GraphPad Prism software (version 10). The ROUT method (*Q* = 1%) was used for identifying outliers. For single comparisons, 2-tailed unpaired Student’s *t* test was performed. For multiple comparisons, 1-way ANOVA with Tukey’s multiple comparisons test was performed. Error bars indicate the SEM. *P* values < 0.05 were considered statistically significant.

### Study approval.

This study complies with all ethical regulations defined by the Institutional Animal Care and Use Committee (IACUC) and Institutional Biosafety Committee of The Ohio State University in Columbus, Ohio, USA (IACUC approval: 2019A00000107-R1). Animals were housed in the University Laboratory Animal Resources Health Sciences complex at The Ohio State University in rodent barrier housing utilizing individually ventilated caging systems. All animals used in this study were humanely euthanized via CO_2_ inhalation.

### Data availability.

Published RNA-Seq (GSE203065) and ATAC-Seq (GSE203064) data sets were analyzed and used in this study. The following publicly available ChIP-Seq data were obtained from ChIP Atlas (https://chip-atlas.org/) for use in this study: STAT1 (GSM994528), STAT4 (GSM550303), T-bet (GSM836124), and Aiolos (GSM5106065). Values for all data points in graphs are reported in the Supporting Data Values file.

## Author contributions

MRL assisted with the design of the study, performed experiments, analyzed data, and wrote the manuscript. DMJ, KAR, SP, JAT, and RTW assisted with experiments and data analysis. JSY provided reagents for influenza virus infection experiments. KJO supervised the research, designed the study, analyzed data, and edited the manuscript.

## Supplementary Material

Supplemental data

Unedited blot and gel images

Supporting data values

## Version 1 11/19/2024

In-Press Preview

## Version 2 01/09/2025

Electronic publication
