# Supplementary material for: Aiolos promotes CXCR3 expression on Th1 cells via positive regulation of IFN-γ/STAT1 signaling
Source: JCI Insight. 2024 Nov 19;10(1):e180287. doi: 10.1172/jci.insight.180287 (PMC11721307; doi:10.1172/jci.insight.180287)
Supplement: Supplemental data [file jciinsight-10-180287-s023.pdf]

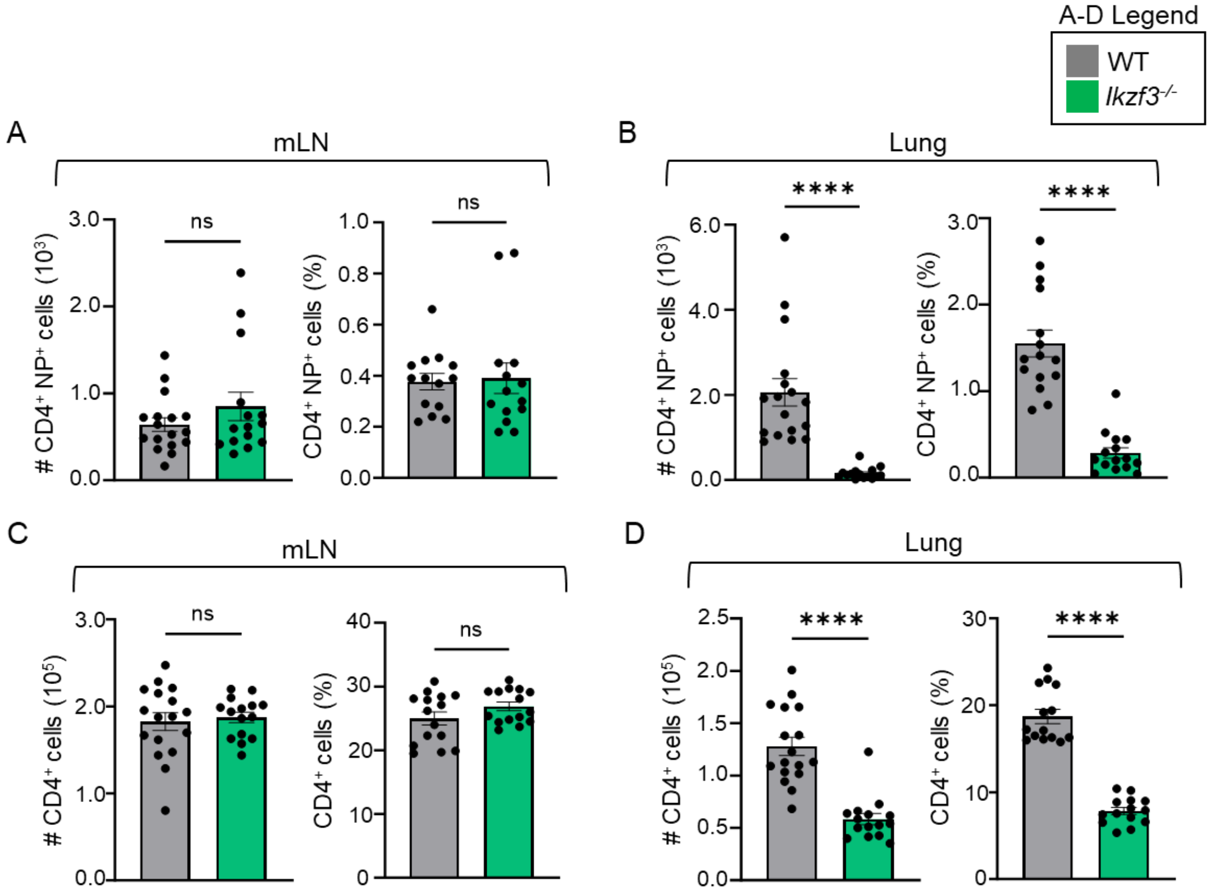

Supplemental Figure 1. Aiolos-deficient mice have reduced numbers of CD4^{+} T cells in the lungs during IAV infection. WT or *Ikzf3*$^{-/-}$ mice were infected intranasally with 30 PFU of IAV (A/PR/8/34; "PR8"). After 8 days, mLN and lungs were harvested and stained for flow cytometric analysis. Fluorochrome-labeled MHC II tetramers were used to identify IAV nucleoprotein (NP)-specific CD4^{+} T cells. **A-B)** NP-specific CD4^{+} T cells were enumerated. Cell numbers were normalized to 1 x 10^6 total events. Numbers and percentages of NP-specific CD4^{+} T cells in the mLN and lungs are displayed. Data from 4 independent experiments is shown (For cell numbers, n = 17 for WT and n = 15 for *Ikzf3*$^{-/-}$. For percentages, n = 14 for mLN and n = 15 for lungs. Data are presented as mean ± SEM; ****p<0.0001, two-tailed unpaired Student's t test). **C-D)** Bulk CD4^{+} T cells were enumerated. Cell numbers were normalized to 1 x 10^6 total events. Numbers and percentages of bulk CD4^{+} T cells in the mLN and lungs are displayed. Representative data from 4 independent experiments shown (For cell numbers, n = 17 for WT and n = 15 for *Ikzf3*$^{-/-}$. For percentages, n = 15 for mLN and n = 14 for lungs. Data are presented as mean ± SEM; ****p<0.0001, two-tailed unpaired Student's t test).

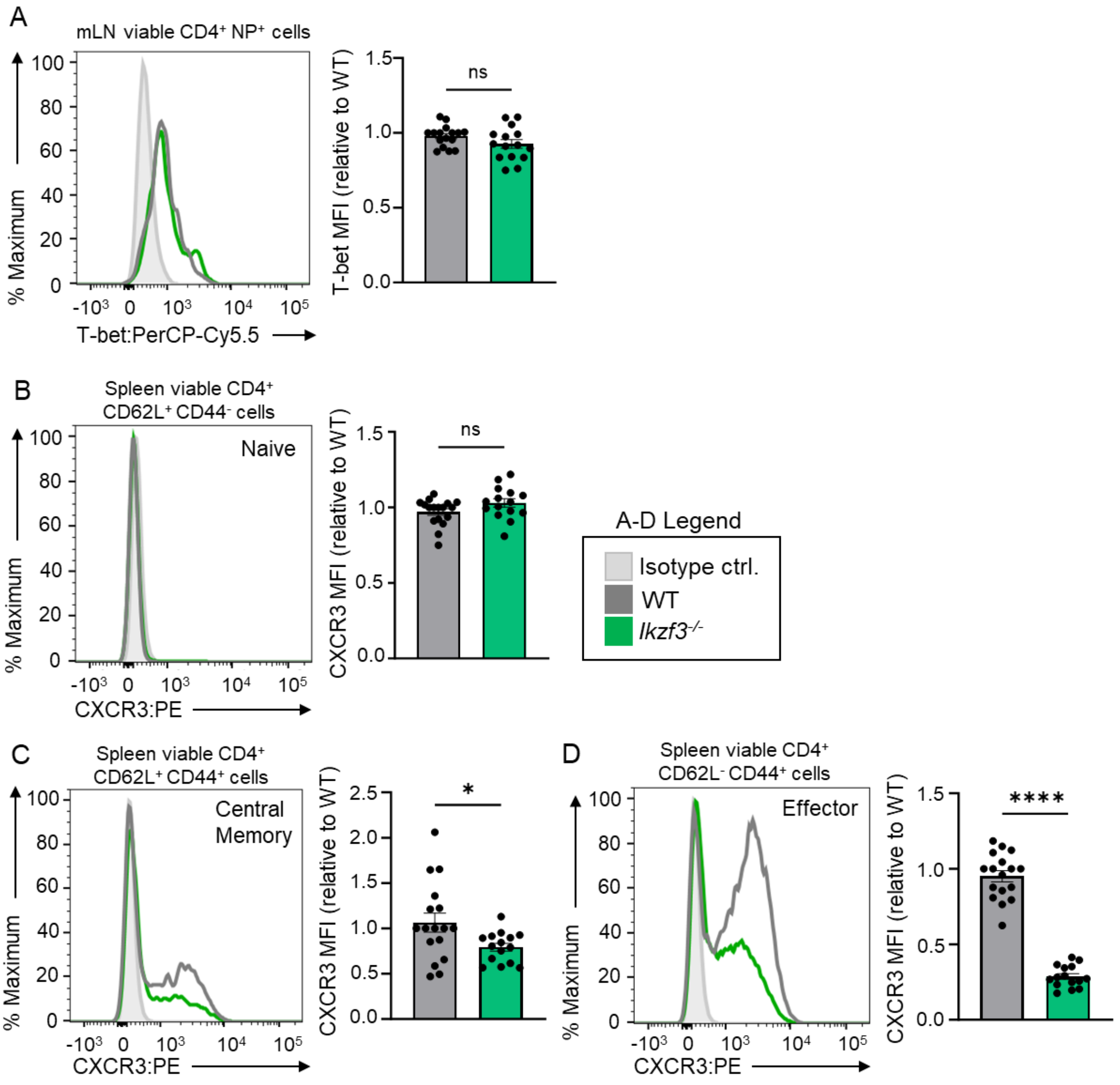

Supplemental Figure 2. Aiolos-deficient mice exhibit no change in T-bet expression but have decreased CXCR3 expression during IAV infection. WT or *Ikzf3*$^{-/-}$ mice were infected intranasally with 30 PFU of IAV (A/PR/8/34; "PR8"). After 8 days, mLN and spleen were harvested and stained for flow cytometric analysis. Fluorochrome-labeled MHC II tetramers were used to identify IAV nucleoprotein (NP)-specific CD4^{+} T cells in the mLN. **A)** Representative flow cytometric analysis for T-bet expression in NP-specific cells isolated from the mLN of WT or *Ikzf3*$^{-/-}$ mice. Data are compiled from 4 independent experiments and displayed as MFI fold change compared to WT controls (n = 16 for WT and n = 15 for *Ikzf3*$^{-/-}$, mean ± SEM; two-tailed unpaired Student's t test). **B-D)** Representative flow cytometric analyses for CXCR3 expression in bulk CD4^{+} naïve (CD62L^{+}CD44^{-}), central memory (CD62L^{+}CD44^{+}), and effector (CD62L^{-}CD44^{+}) T cell populations isolated from the spleens of WT or *Ikzf3*$^{-/-}$ mice. Data are compiled from 4 independent experiments and displayed as MFI fold change compared to WT controls (n = 17 for WT and n = 15 for *Ikzf3*$^{-/-}$, mean ± SEM; *p<0.05, ****p<0.0001, two-tailed unpaired Student's t test).

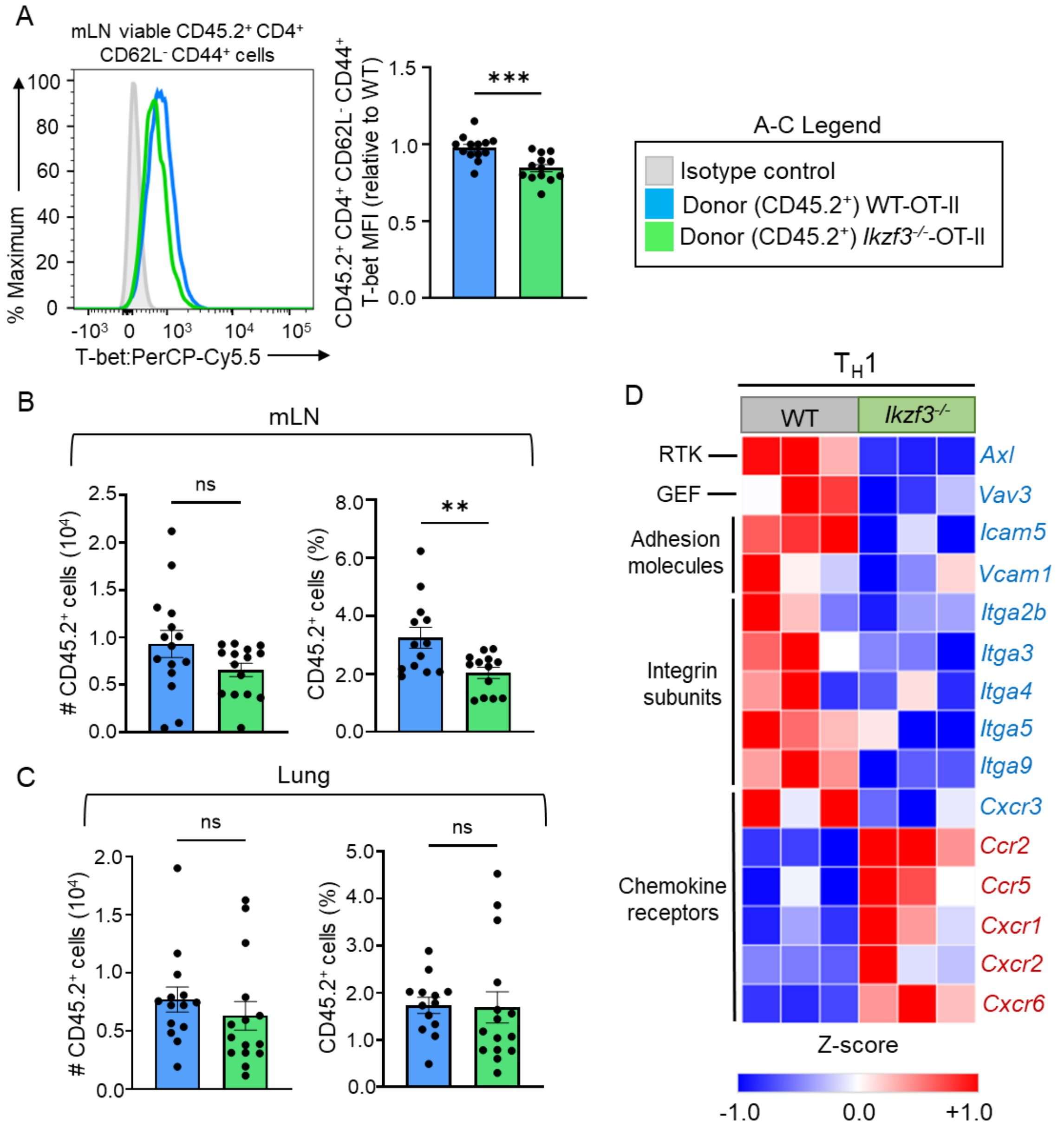

Supplemental Figure 3. CD4⁺ T cells display altered migratory programs in the absence of Aiolos. Naïve CD4⁺ T cells were harvested from the mLN and lungs of WT-OT-II or *Ikzf3*$^{-/-}$-OT-II mice. 500,000 cells/animal were adoptively transferred into CD45.1⁺ recipients. Recipient mice were then infected with 40 PFU of $OVA_{323–339}$-expressing A/PR/8/34 ("PR8-OVA") influenza virus 24 hours post-transfer. 8 days post-infection, mLN and lungs were harvested and viable $CD45.2^+CD4^+CD62L^-CD44^+$ (antigen-specific, donor effector) cells were analyzed via flow cytometry. **A)** Representative flow cytometric analysis for T-bet expression in $CD45.2^+CD4^+CD62L^-CD44^+$ cells in the mLN. Data are compiled from 3 independent experiments and displayed as MFI fold change compared to WT-OT-II control cells (n = 13, mean ± SEM; ***p<0.001, two-tailed unpaired Student's t test). **B-C)** Total $CD45.2^+$ cell numbers were enumerated. Cell numbers were normalized to 500,000 total events. Numbers and percentages of $CD45.2^+$ cells in the mLN and lungs are displayed. Data from 3 independent experiments is shown (For cell numbers, n = 14-15. For percentages, n = 13-15. Data are presented as mean ± SEM; **p<0.01, two-tailed unpaired Student's t test). **D)** Published RNA-seq data (GSE203065) from in vitro-generated WT and *Ikzf3*$^{-/-}$ T_H1 cells was analyzed for differentially expressed genes (DEGs). A heatmap of DEGs associated with cell migration in T_H1 cells is shown. Gene names color-coded in blue are downregulated in *Ikzf3*$^{-/-}$ T_H1 cells. Gene names color-coded in red are upregulated in *Ikzf3*$^{-/-}$ T_H1 cells. Note: *Cxcr3* transcript data presented here is the same as in Figure 4B. RTK; receptor tyrosine kinase. GEF; guanine nucleotide exchange factor.

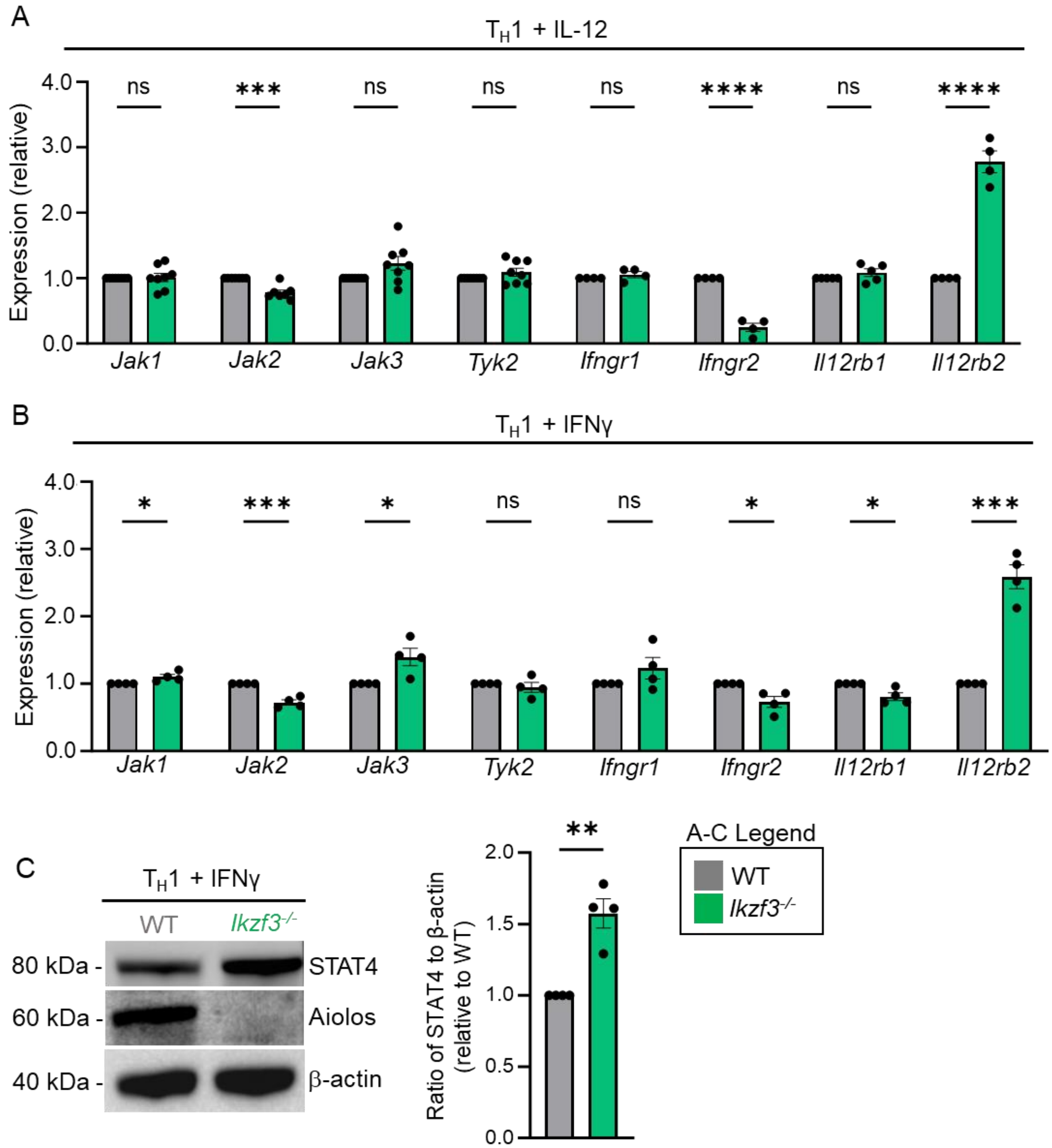

Supplemental Figure 4. IFNγ/STAT1 and IL-12/STAT4 pathways are altered in Aiolos-deficient T_H1 cells. Naïve $CD4^+$ T cells were harvested from WT and *Ikzf3*$^{-/-}$ mice and stimulated with α-CD3/CD28 under T_H1 polarizing conditions (IL-12, α-IL-4). On day 3, cells were removed from stimulation and given either 1.) IL-12, α-IL-4, and IL-2 or 2.) IFNγ, α-IL-4, and IL-2 for an additional 2 days prior to harvest. **A)** At day 5, transcript analysis was performed on IL-12-treated T_H1 cells via qRT-PCR. Transcript was normalized to *Rps18* and presented as fold change compared to WT control (n = 4-8 biological replicates from 4-8 independent experiments. Data are presented as mean ± SEM; ***p<0.001, ****p<0.0001, two-tailed unpaired Student's t test). **B)** At day 5, RNA was isolated from IFNγ-treated T_H1 cells, and transcript analysis was performed as in 'A' (n = 4 biological replicates from 4 independent experiments, mean ± SEM; *p<0.05, ***p<0.001, two-tailed unpaired Student's t test). **C)** An immunoblot of IFNγ-treated T_H1 cells was performed to assess the relative abundance of the indicated proteins. β-actin serves as a loading control (n = 4 independent experiments, mean ± SEM; **p<0.01, two-tailed unpaired Student's t test).

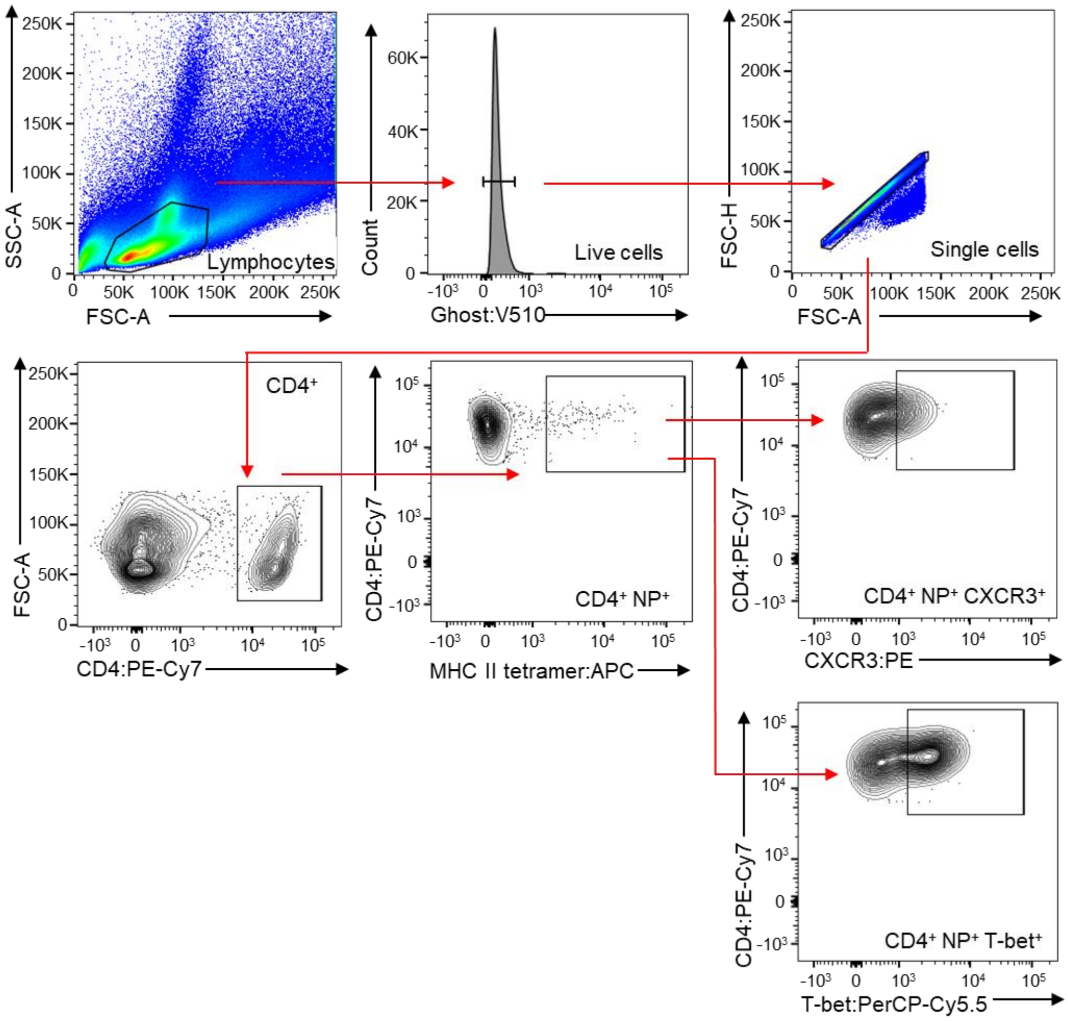

Supplemental Figure 5. Representative flow cytometry gating strategy for mediastinal lymph node (mLN) and lungs in germline knockout IAV infection experiments. WT or *Ikzf3*$^{-/-}$ mice were infected intranasally with 30 PFU of IAV (A/PR/8/34; "PR8"). After 8 days, mLN and lungs were harvested and stained for flow cytometric analysis of CXCR3 and T-bet expression in IAV nucleoprotein (NP)-specific CD4^{+} T cells. Fluorochrome-labeled MHC II tetramers were used to identify NP-specific cells in the mLN and lungs.

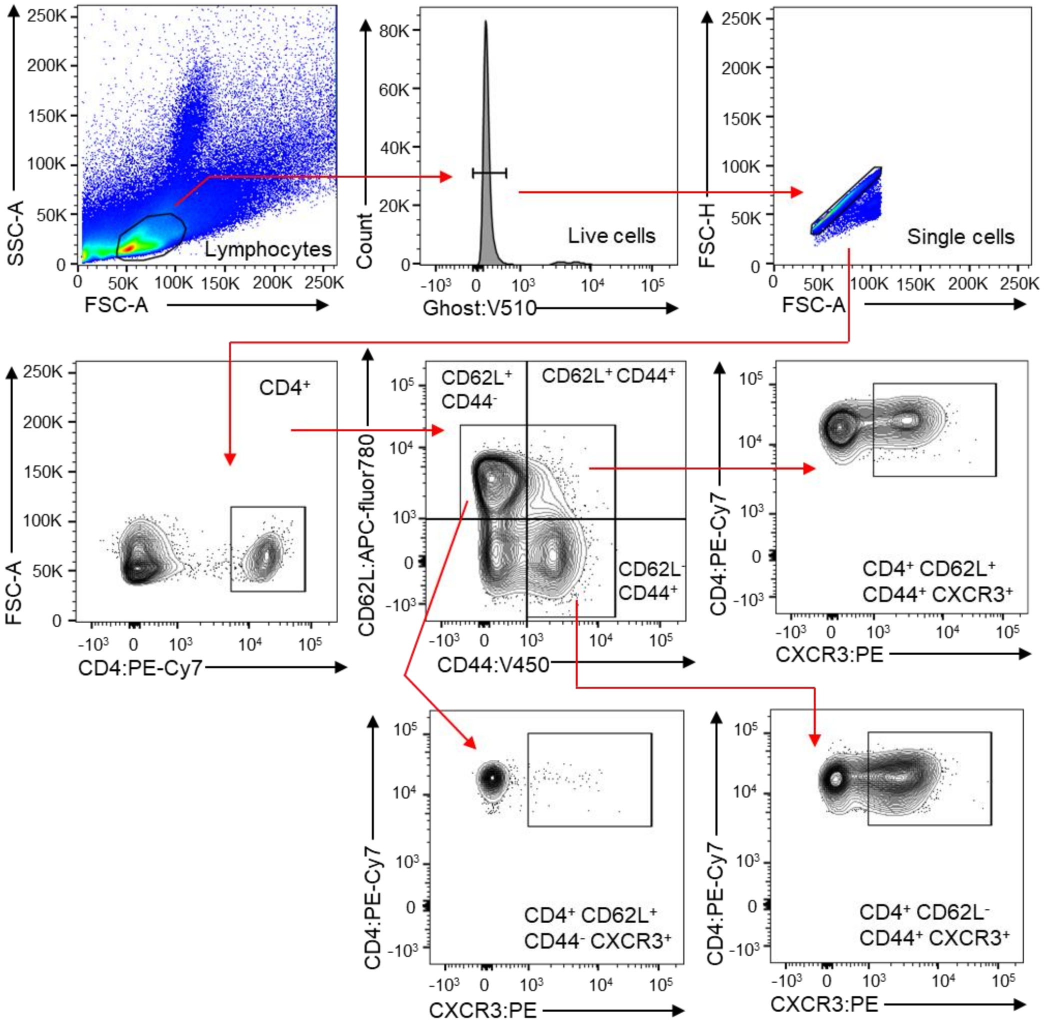

Supplemental Figure 6. Representative flow cytometry gating strategy for spleen in germline knockout IAV infection experiments. WT or *Ikzf3*$^{-/-}$ mice were infected intranasally with 30 PFU of IAV (A/PR/8/34; "PR8"). After 8 days, spleen was harvested and stained for flow cytometric analysis of CXCR3 expression in bulk CD4$^+$ naïve (CD62L$^+$CD44$^-$), central memory (CD62L$^+$CD44$^+$), and effector (CD62L$^-$CD44$^+$) T cell populations.

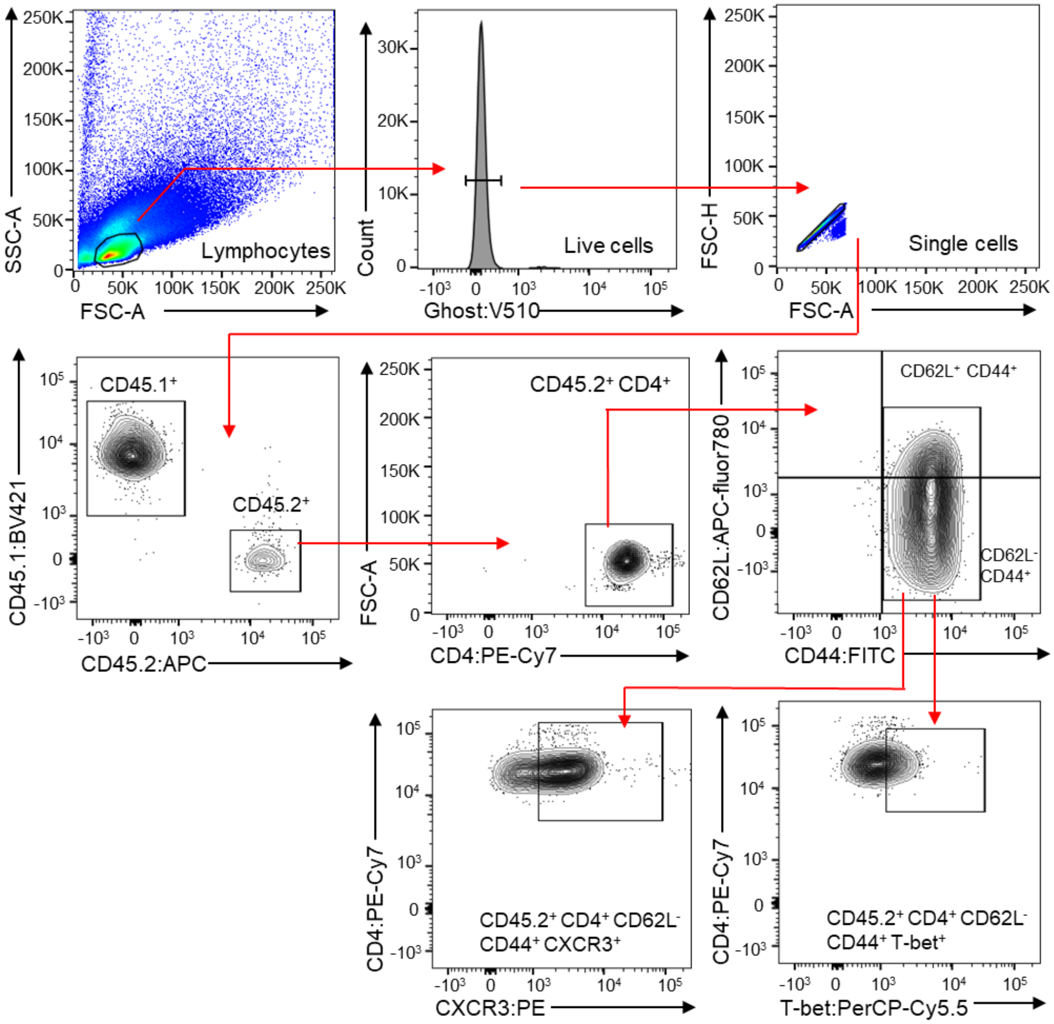

Supplemental Figure 7. Representative flow cytometry gating strategy for adoptive transfer experiments. Naïve CD4^{+} T cells were harvested from the mLN of WT-OT-II or *Ikzf3*$^{-/-}$-OT-II mice. 500,000 cells/animal were adoptively transferred into CD45.1^{+} recipients. Recipient mice were then infected with 40 PFU of OVA$_{323–339}$-expressing A/PR/8/34 (“PR8-OVA”) influenza virus 24 hours post transfer. 8 days post-infection, mLN was harvested and viable CD45.2^{+}CD4^{+} CD62L^{-}CD44^{+} (antigen-specific, donor effector) cells were analyzed via flow cytometry for CXCR3 and T-bet expression.

Supplemental Table 1. qRT-PCR primers

Gene (murine)	Forward (5'-3')	Reverse (5'-3')
Rps18	GGAGAACTCACGGAGGATGAG	CGCAGCTTGTTGTCTAGACCG
Ikzf3	CCGACTGTGGAGCTGAAAAGC	CCTGCATCTTCGTCTTCATTGG
Cxcr3	CCTTGAGGTTAGTGAACGTC	GCTGGCAGGAAGGTTCTGTC
Tbx21	GTGACTGCCTACCAGAACGC	AGGGGACACTCGTATCAACAG
Stat1	GGTACAACATGCTGGTGACAGAG	CTCCCAGCATGCTCAGCTGGTC
Stat4	CCAATGGGAGCCTCTCAGTGGAG	GCAACTCCTCTGTCACCATGTG
Jak1	GCTGAGGTGGAGCTGCACCGAC	GTCCATAGAGCCATGCAGGCTG
Jak2	GGAAACTTGGAGTGGCTAAGCAG	GTGGGTTCCCCGTTCTCCTGTC
Jak3	CCTGATCTGCGACTCCAGGC	GAGAATGTAGGTGCCTGGGAG
Tyk2	GGAGCGTCGCGTGCACATCCAC	GTGGCTGGAGTCAGCAGTCAAGC
Ifngr1	GTGTATGTGGAGCATAACCGGAG	CTGGAATCCAGTGTGGATACTGAG
Ifngr2	GAGCAATGTATCCTGTCACG	GTCAGGCCGAGCAGCAATGCG
Il12rb1	CACGACTCGGCTCCTCATGGAC	TCTCAACGCAGCCATCACC
Il12rb2	CTTGGACGGCATCAGTGTCTGC	GACCTGGTGAGGAGCCAGCAAC

Supplemental Table 2. Promoter-reporter primers

Gene (murine)	Forward (5'-3')	Reverse (5'-3')
***Stat1* prom.**	GATCGGTACCGCAGGCTTGGTTGACGTCAGTG	GATCGAGCTCAGGGCGTCCCGCCTCCTTCCGCCTC

Supplemental Table 3. ChIP qPCR primers

Gene (murine)	Forward (5’-3’)	Reverse (5’-3’)
***Cxcr3* prom.**	CAGGTCTCGTGCTGCCTGCTTCTC	CTGCGGAGGGCTGGTATAGATTACC
***Cxcr3* enhc.**	GGGAGAAAGTGACAGTGCAG	CAGACATTAGCATGAAGCCACC
***Cxcr3* ctrl.**	GCCTAGGGAAGATAGTTCCTC	GGTTGAAGCAGGGAGTGGTGG
***Ikzf3* prom.**	GACGTCTACTTGAGAAACACCGG	CACTGACAGTTCTCAAGACCGTC
***Ikzf3* ctrl.**	GTGCAGCTTCCCAATAAACCTGCC	GGAACTCACCATGTAGACCAGGCTG

Supplemental Table 4. BioRender Publication Licenses

Figure	Citation
**Graphical abstract**	Leonard, M. (2024) https://BioRender.com/i42i899
**Figure 1B**	Leonard, M. (2024) https://BioRender.com/v70c175
**Figure 2A**	Leonard, M. (2024) https://BioRender.com/p22p915
**Figure 3A**	Leonard, M. (2024) https://BioRender.com/h43w136
**Figure 4C**	Leonard, M. (2024) https://BioRender.com/b87z682
**Figure 5A**	Leonard, M. (2024) https://BioRender.com/i17z581
**Figure 6A**	Leonard, M. (2024) https://BioRender.com/u39m539
**Figure 7A**	Leonard, M. (2024) https://BioRender.com/r22y787
