# Supplementary material for: Aiolos promotes CXCR3 expression on Th1 cells via positive regulation of IFN-γ/STAT1 signaling
Source: JCI Insight. 2024 Nov 19;10(1):e180287. doi: 10.1172/jci.insight.180287 (PMC11721307; doi:10.1172/jci.insight.180287)

Full unedited Western Blot Images for Figure 5C

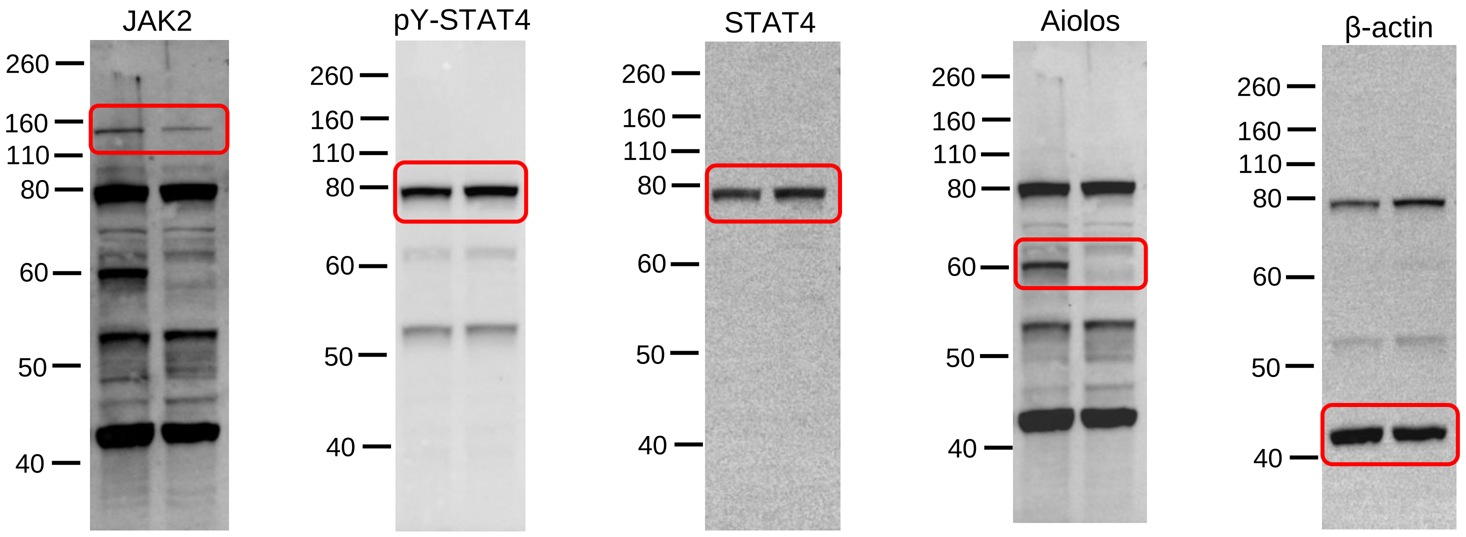

Full unedited Western Blot Images for Figure 5D

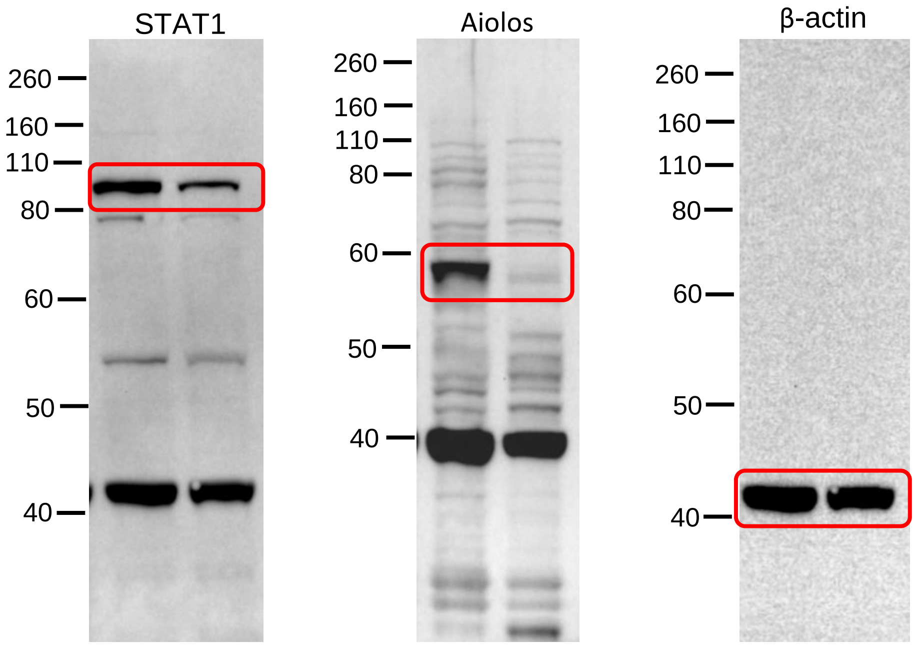

Full unedited Western Blot Images for Figure 6D

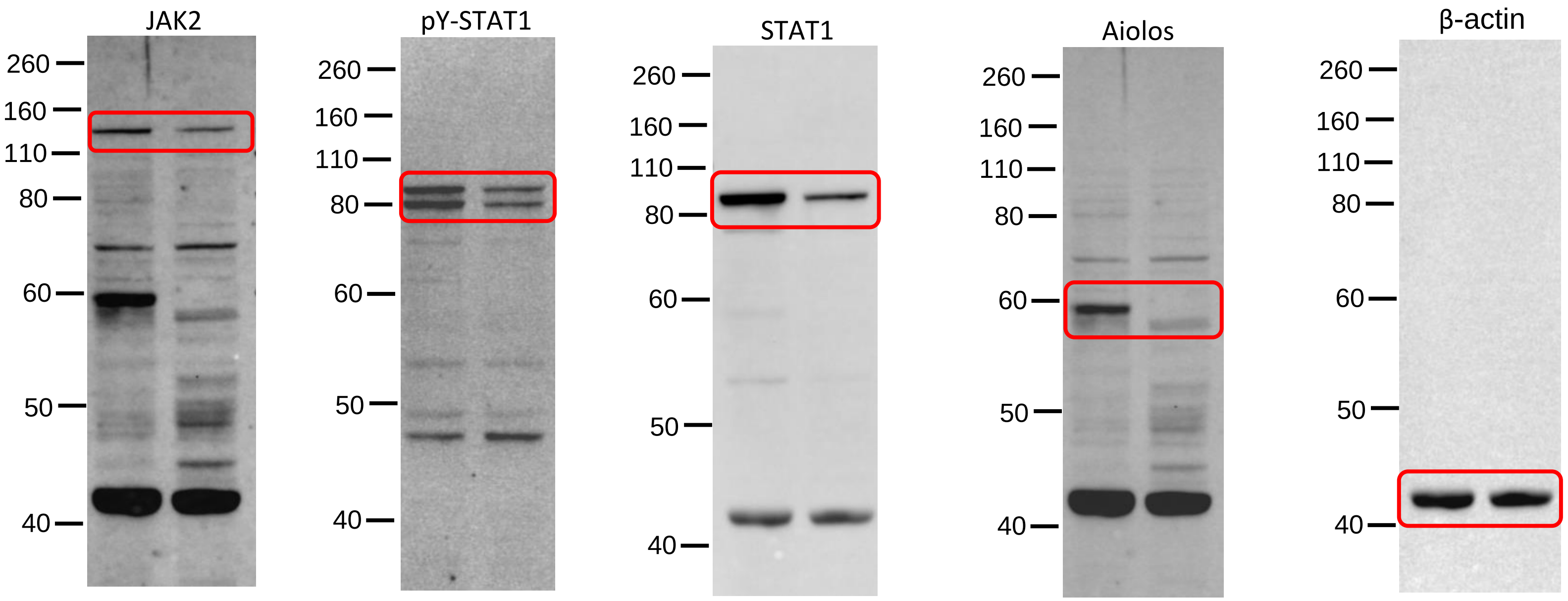

Full unedited Western Blot Images for Figure 7D

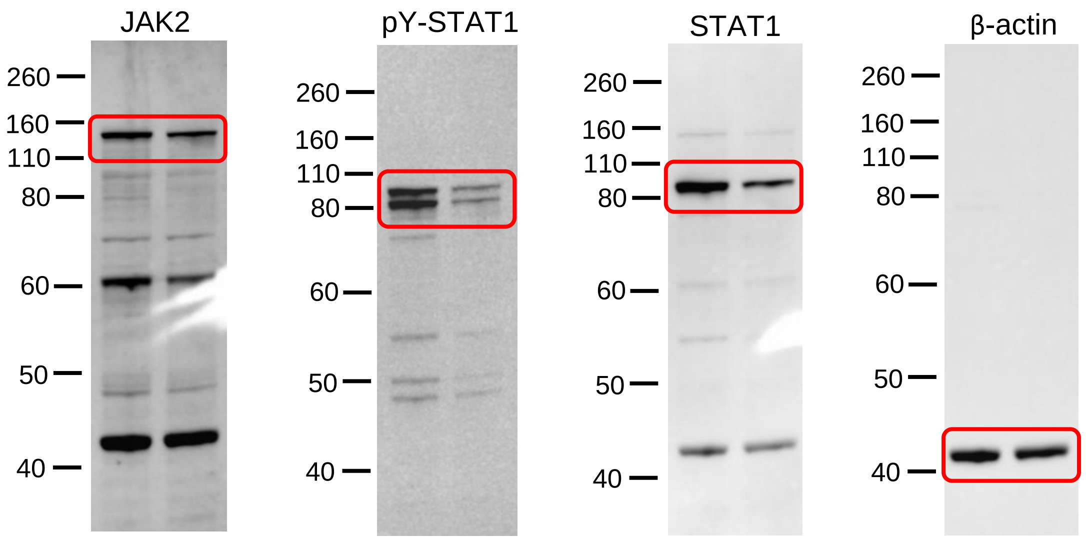

Full unedited Western Blot Images for Figure 8C

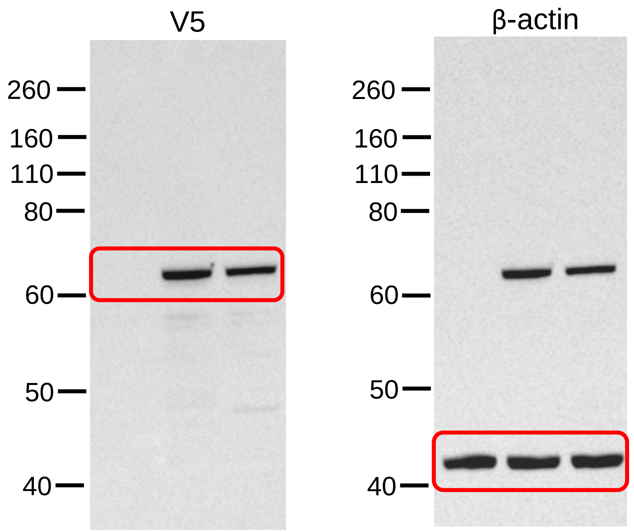

Full unedited Western Blot Images for Supplemental Figure 4C

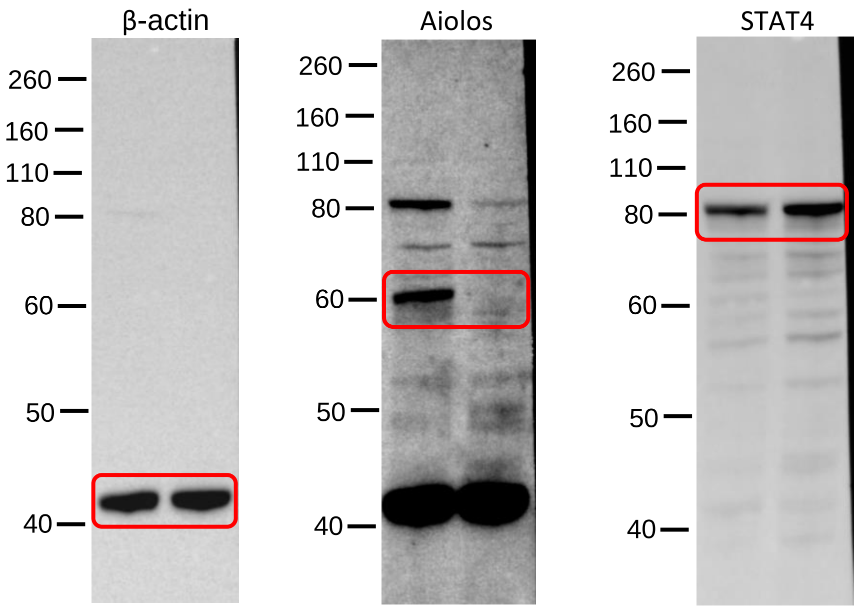

Supplement: Unedited blot and gel images [file jciinsight-10-180287-s024.pdf]
